# A randomized, double-blind, placebo-controlled, multicentre trial on the efficacy of varenicline and bupropion in combination and alone for treatment of alcohol use disorder: Protocol for the COMB study

**DOI:** 10.1371/journal.pone.0296118

**Published:** 2024-01-11

**Authors:** Andrea de Bejczy, Helga Lidö, Bo Söderpalm

**Affiliations:** 1 Addiction Biology Unit, Psychiatry and Neurochemistry Section, Institute of Neuroscience and Physiology, Sahlgrenska Academy, University of Gothenburg, Gothenburg, Sweden; 2 Department of Addiction and Dependency, Sahlgrenska University Hospital, Gothenburg, Sweden; University of British Columbia, CANADA

## Abstract

**Background:**

Alcohol Use Disorder (AUD) is a major cause of premature death, disability and suffering. Available treatments are of modest efficacy and under-prescribed so there is a pressing need for a well-tolerated and effective treatment option for AUD. Dopamine is hypothesized to be involved in the development of alcohol dependence. To challenge the low-dopamine hypothesis of addiction, this randomized, double-blind, placebo-controlled, 13-week, multicentre clinical trial with four parallel arms is designed to evaluate the efficacy of two substances raising dopamine levels, varenicline and bupropion, alone and in combination vs. placebo on alcohol consumption in AUD. Varenicline, a partial agonist at brain nicotinic acetylcholine receptors increases dopamine release, whereas bupropion is a centrally-acting, norepinephrine-dopamine reuptake inhibitor. Varenicline is previously shown to reduce alcohol intake in individuals with AUD. We hypothesize that the effect size of a combination of two drugs affecting dopamine levels in the brain will exceed that of approved AUD therapies.

**Methods:**

Consenting individuals with AUD will be recruited via media advertisements. Those fulfilling the eligibility criteria (N = 380) will be randomized to one of four interventions (n = 95 per arm). Treatment will comprise one week of titration (varenicline 0.5‒2 mg; bupropion SR 150‒300 mg) plus 12 weeks at steady state. Efficacy will be evaluated using two primary endpoints of alcohol consumption: Heavy Drinking Days and blood levels of phosphatidylethanol. Secondary objectives, exploratory and subgroup analyses will be also performed. The modified Intention-to-Treat and Per Protocol datasets will be evaluated using Analysis of Covariance. Last patient out is estimated to occur in December, 2022.

**Discussion:**

The COMB Study aims to evaluate the efficacy of the combination of varenicline and bupropion, two drugs affecting dopamine, on alcohol consumption, and to challenge the low-dopamine hypothesis of addiction. Study Code COMB-BO8, EudraCT 2018–000048–24, Version 3.2, Lidö & deBejczy, 2020-06-16; https://clinicaltrials.gov identifier NCT04167306.

## Introduction

Alcohol is a major contributor to the global burden of disease [1], and the average lifetime expectancy of individuals with alcohol use disorder (AUD) is estimated to be shortened by 23 years [2]. The lifetime risk of death due to alcohol-related causes (accidents and diseases) increases exponentially with alcohol consumption [3], and both abstinence and the reduction of alcohol intake can profoundly lower the risk of death in the heaviest consumers [4]. While many individuals with AUD are not motivated by the abstinence goal, they can accept the low-risk drinking level goal [5, 6]. Indeed, focus in alcohol treatment trials has shifted from abstinence as a goal to one of reducing alcohol consumption to lower risk levels [7, 8].

Currently, four medications are available for AUD: disulfiram, acamprosate, naltrexone, and nalmefene. Disulfiram is an aversion therapy with a good short-term effect albeit only in individuals who accept the abstinence goal [9]. In contrast, acamprosate, naltrexone, and nalmefene act by reducing the reinforcing effects of and/or craving for alcohol, and can be used for both abstinence and harm reduction goals [10]. Nevertheless, their effect sizes are small (Cohen’s d = 0.2) [11–13], and all four are under-prescribed. Consequently, the treatment gap has been estimated to be over 90% [14] so new treatment options with larger effect sizes could greatly enhance treatment rates for AUD. Notably, neither disulfiram, naltrexone nor acamprosate was developed based on the neurobiology of AUD.

Alcohol interferes with the dopaminergic brain reward system. The dopamine hypothesis of drug intake and addiction focused initially on the acute rewarding, stimulative, and positive reinforcing effects of drugs of abuse, including ethanol, which appear to involve enhanced dopamine release in the nucleus accumbens (ventral striatum in man) [15, 16]. Blocking these positive reinforcing effects with pharmacological agents, therefore, was hypothesized to eliminate drug taking behaviour, which is also the assumed mechanism of action for naltrexone that at least partly prevents the dopamine releasing effect of ethanol [17]. Later, the dopamine hypothesis also included the low-dopamine state that develops upon chronic exposure to addictive drugs, and which is hypothesised to drive alcohol intake by negative reinforcement [18].

The discovery that the dopamine releasing effect of alcohol in the nucleus accumbens involves nicotinic acetylcholine receptors (nAChRs) located in the ventral tegmental area in the midbrain [19–21] triggered animal [22] and human experimental studies [23], as well as randomized controlled trials (RCTs) to investigate the use of varenicline in AUD [24, 25]. Varenicline, indicated for smoking cessation, is a selective, partial agonist of the nAChR of subtype conformation α_4_β_2_ that elevates extracellular dopamine levels in the nucleus accumbens [26], probably by stimulating α_4_β_2_ nAChRs located on dopaminergic cell bodies in the ventral tegmental area and/or on dopaminergic terminals in the nucleus accumbens [27]. In RCTs, varenicline has been shown to reduce alcohol intake with an effect size of approx. 0.35‒0.4 (Cohen’s d) [24, 25].

Although an effect size of 0.4 is larger than that for acamprosate and naltrexone, even higher effect sizes would be desirable. Treatments for comparable psychiatric public health issues, e.g. depression and attention-deficit/hyperactivity disorder (ADHD), offer effect sizes between 0.5‒0.8 [28, 29]. By combining two agents with complimentary mechanisms of action, we aim for an effect size larger than that of currently available treatments for AUD.

To complement the dopamine action of varenicline, we have chosen bupropion, an approved treatment for smoking cessation and depression. This centrally-acting norepinephrine-dopamine reuptake inhibitor elevates brain levels of norepinephrine and dopamine by blocking the action of both the norepinephrine and dopamine transporters with minimal affinity for the serotonin transporter [30]. Interestingly, in a recent preclinical study we showed that the combination of varenicline and bupropion enhanced dopamine release in rat nucleus accumbens and was associated with a synergistic, abolishing action on the alcohol deprivation effect in high alcohol-consuming rats [31], a measure considered to have a high predictive value for effects in humans [32].

Our study (COMB-BO8; NCT04167306; aka the COMB Study, full protocol can be assessed in S5 File and SPIRIT checklist in S1 File), therefore, has been designed to test the hypothesis that varenicline and bupropion, either alone or in combination, will reduce alcohol consumption in individuals with AUD. Varenicline is hypothesized to reduce alcohol consumption measured by blood phosphatidylethanol (B-PEth) levels and heavy drinking days (HDD) compared to baseline and significantly more than placebo with an expected effect size (B-PEth) of approximately 0.35 (Cohen’s d). Combination treatment with varenicline and bupropion is hypothesized to reduce B-PEth levels and HDD compared to baseline and significantly more than placebo with an effect size (B-PEth) of 0.5‒0.6 (Cohen’s d).

In summary, the COMB study is a Phase II, randomized double-blind, placebo-controlled, 13-week, multicentre trial with four parallel groups designed to assess the efficacy and safety of varenicline and bupropion alone and in combination vs. placebo for alcohol reduction in alcohol-dependent subjects, and to challenge the low-dopamine hypothesis of alcohol addiction.

## Methods

### Study setting

The study will be conducted at four alcohol clinic research sites in Sweden: Gothenburg (Sponsor and Coordinating Centre), Stockholm, Linköping, and Region Skåne. The study sites and principal investigators are listed in S1 Table. A central laboratory appointed by the Coordinating Centre will analyse B-PEth (both a primary outcome and an eligibility measure) from all study sites. All safety parameters and secondary outcome measures (described in “Measures”) will be analysed at each site according to site-specific practice.

The study period comprises a total of nine visits to a research site; one screening visit to assess eligibility followed, within 10 days, by randomization, 1 week of titration; and, bi-weekly visits until the end of treatment by Visit 9. Subjects will be followed up for safety on days 7 and 30 by phone call. Treatment duration comprises 1 week of titration plus 12 weeks of steady-state treatment.

### Screening and eligibility criteria

#### Inclusion criteria

Subjects aged 25‒70 years at screening and diagnosed with moderate and severe AUD according to DSM-V (meeting ≥ 4 out of 11 criteria) must provide signed informed consent and have a blood alcohol level < 0.1‰ (0.1 g/L) at signing. Subjects must also have B-PEth levels of ≥ 0.5 μmol/L at screening (Visit 1) and continuous, high alcohol consumption over the last 3 months prior to screening (defined by ≥ 2 HDD per week during a typical week). They should also be available for contact by phone, and be able to read and write in Swedish.

#### Exclusion criteria

Subjects with total abstinence between the screening and randomization visits will be excluded, as will those who received alcohol withdrawal treatment within 30 days of study initiation or any pharmacological (including, but not exclusive to, varenicline, bupropion, disulfiram, acamprosate, naltrexone, nalmefene, baclofen, topiramate, ondansetron, mirtazapine, methylphenidate, dexamphetamine, atomoxetine, pregabalin, buprenorphine, and methadone) or non-pharmacological treatment affecting alcohol consumption within 3 months of study initiation and during the study period that could affect alcohol consumption. Subjects using antidepressants, opioid analgesics, benzodiazepines, zopiclone, zolpidem, hydroxizine, alimemazine, propiomazine or other sedatives continuously, but not sporadically, will be excluded, as well as those using any concurrent medication that may affect the results of the trial or compromise the safety of the subjects (the SmPCs for any concurrent medication will be checked for possible interactions with study drugs).

Subjects with laboratory hepatic values > 3 times the upper limit of the normal range, creatinine clearance < 30 ml/min or other clinically significant abnormalities in the screening clinical laboratory values; and/or, blood pressure ≥ 180/110 at screening will be excluded, as will any pregnant or breast-feeding subject or premenopausal female not using the allowed contraceptive methods (oral contraceptive, copper/hormonal intrauterine contraceptive device or subcutaneous implants).

Subjects with diabetes mellitus types 1 or 2 who require insulin treatment; any current psychiatric or somatic disorder or condition that may affect assessments or compromise their safety; Adult ADHD Self-Report Scale (ASRS; v. 1.1) Part A score ≥ 4 in the marked cut-off section; Montgomery Åsberg Depression Rating Scale (MADRS) score ≥ 20; current, non-mild depression; suicidality; current illicit drug use based on urine-toxicity testing and a Drug Use Disorder Identification Test (DUDIT); a history of delirium tremens or abstinence-induced seizures within 5 years of study initiation; non-alcohol-induced epilepsy/seizures (lifetime); severe sleep disturbances; a need for alcohol detoxification; living conditions not appropriate to fulfil study requirements; herbal drugs/tea and supplementations use that might affect outcome(s) or safety; previous randomization in this trial or participation in another trial within 3 months of enrolment into this trial; and, additional factors that render the subject unable to complete the study (as judged by the investigator) will be excluded.

No concomitant care for AUD will be allowed, although somatic care will be allowed if this includes no prohibited medications. Non-eligible subjects will be referred to an appropriate alcohol clinic/healthcare center or, if required due to a medical condition other than alcohol dependence or an alcohol-related condition, may be referred to an appropriate clinic.

### Interventions, dosages, and administration

Varenicline tartrate (Pfizer Innovations, USA) will be used to manufacture varenicline, the investigational medicinal product (IMP) 1. The dose of varenicline will be escalated from 0.5 to 2 mg daily during the first week as follows: 0.5 mg varenicline (or corresponding placebo capsules) once daily on days 1‒3 and 0.5 mg varenicline (or corresponding placebo capsules) twice daily on days 4‒7, after which 1 mg varenicline (or corresponding placebo capsules) will be administered twice daily (1 mg x 2 p.o. daily) on days 8‒91 of treatment (visits 3‒9). No dose adjustment of varenicline will be allowed.

Bupropion Sandoz SR (Slow Release formulation; Sandoz AS, Switzerland) will be used to manufacture bupropion (IMP 2). The dose of bupropion will be escalated from 150 to 300 mg daily during the first week as follows: bupropion SR 150 mg or corresponding placebo capsules once daily on days 1‒7, after which 150 mg bupropion SR or corresponding placebo capsules will be administered twice daily (150 mg x 2 p.o. daily) on days 8‒91 of treatment (visits 3‒9). No dose adjustment of bupropion will be allowed.

Apoteket Produktion & Laboratorier (APL) will manufacture the IMPs using a procedure to encapsulate tablets, and package and label them according to Good Manufacturing Practice (GMP). The IMPs will be packaged in unit-dose blister packs and distributed according to GMP. The formulation of the IMPs and placebo will be indistinguishable as regards appearance, shape, smell, and taste.

Subjects will receive varenicline or bupropion SR on seven occasions (visits 2, 3, 4, 5, 6, 7 and 8). Treatment will start the day after randomization and after the first week of titration, varenicline and bupropion SR will be distributed to subjects every second week.

As reported previously [24, 25], 12 weeks of steady-state treatment duration are estimated to be sufficient to detect tentative efficacy effects (measured by B-PEth and HDD) whilst concurrently manageable for study participants, thereby also optimizing study compliance.

Adherence strategies and procedures for monitoring adherence to protocol include the return of IMP and blister packs by subjects, pill count, and IMP plasma concentration analysis at visits 4 and 6.

### Study discontinuation

Trial participation is voluntary and subjects can choose to discontinue at any point. The exclusion criteria apply throughout the entire study period and subjects may be withdrawn from the study at the discretion of the investigator if judged non-compliant with study procedures or for safety concerns. The treatment code will not be broken except in medical emergencies when appropriate management of the subject will necessitate knowledge of the treatment randomization.

Should any adverse event (AE) that may affect the benefit-risk balance be reported, subjects may be unblinded to enable a decision to abort the trial. Subjects will be excluded from the analysis set if an AE that is considered to affect the assessments and/or the result of the trial is reported whether or not subject safety is not compromised. Serious adverse events (SAEs) will be followed up.

At pre-term termination, subjects will undergo an end-of-trial assessment.

### Measures

#### Primary objectives and outcomes

The primary objective will be to evaluate the effects of varenicline and bupropion alone and in combination in reducing alcohol consumption in subjects with AUD using two primary efficacy endpoints: (i) alcohol consumption as measured by blood levels (μmol/L) of B-PEth, and (ii) subjective alcohol consumption as measured by HDD using the timeline follow-back (TLFB) procedure and defined as ≥ 5 units for men and ≥ 4 units for women with 1 unit defined as 14 grams of pure alcohol.

Subjective alcohol consumption measured by HDD is a FDA-approved, clinical trial outcome variable for alcohol consumption, whilst the objective alcohol biomarker B-PEth that occurs in red blood cells in humans in the presence of alcohol [33] is a validated biomarker for alcohol consumption. The sensitivity and specificity of B-PEth are 94.5% and 100%, respectively [34], both of which are superior to carbohydrate deficient transferrin (CDT), an indirect alcohol marker for moderate to heavy drinking for approx. 2‒4 weeks [35], and gamma glutamyltransferase (GGT), an indirect alcohol marker for moderate to heavy drinking for approx. 4 weeks [24, 35]. The half-life of B-PEth is approx. 1 week, and the detection window is days to several weeks [36–38].

Blood levels of B-PEth will be calculated as the mean value over the 8-week, steady-state, active treatment period (Visit 4 to Visit 8) vs. baseline. Data will be analysed as the within patient difference. Visits 9 and 3 will be omitted in the analysis period due to the tendency of drop-out and non-compliance at the end of a study period (Visit 9) and due to titration of IMP and the optimal timeframe of B-PEth analysis (Visit 3). Additionally, the B-PEth vs. baseline levels analysis will be performed at all visits.

The HDD number by 14 days is defined as a mean over the 8-week, steady-state, active treatment period (Visit 4 to Visit 8), in 14-day period measures vs. baseline. The baseline mean of HDD by 14 days will be obtained at the screening visit, where the number of HDD over the last 30 days will be recorded. Additionally, the HDD analysis vs. baseline levels will be performed at all visits.

#### Secondary objectives and outcomes

The following secondary objectives will be evaluated: (i) the effects of varenicline and bupropion alone and in combination in reducing alcohol consumption in subjects with AUD using the indirect alcohol markers CDT and GGT, self-reported alcohol consumption using TLFB, the mean grams of alcohol per day, and, the number of drinking days, drinks per drinking days and abstaining days; (ii) the Total score of Alcohol Use Identification Test (AUDIT); (iii) alcohol craving measured by a Visual Analogue Scale (VAS); (iv) nicotine use as measured by the nicotine saliva marker cotinine; (v) the Temporal Experience of Pleasure Scale (TEPS); (vi) the Continuous Performance Test + Activity test (CPTA); (vii) and, the relationships between the aforementioned outcome measures and plasma drug concentrations of bupropion and varenicline.

#### Additional analyses

Efficacy will be evaluated in relation to the plasma drug concentrations of varenicline and bupropion, and for compliance. Subgroup analyses will be performed on the World Health Organization consumption level categories (defined in “Subgroup analyses”), the severity of AUD, and family history of alcohol problems. Data will also be analysed to identify characteristics for responders and to evaluate placebo effects in early responders. Additionally, dopamine-related variables and genetic factors will be analysed.

#### Study assessments

**Fig 1:**
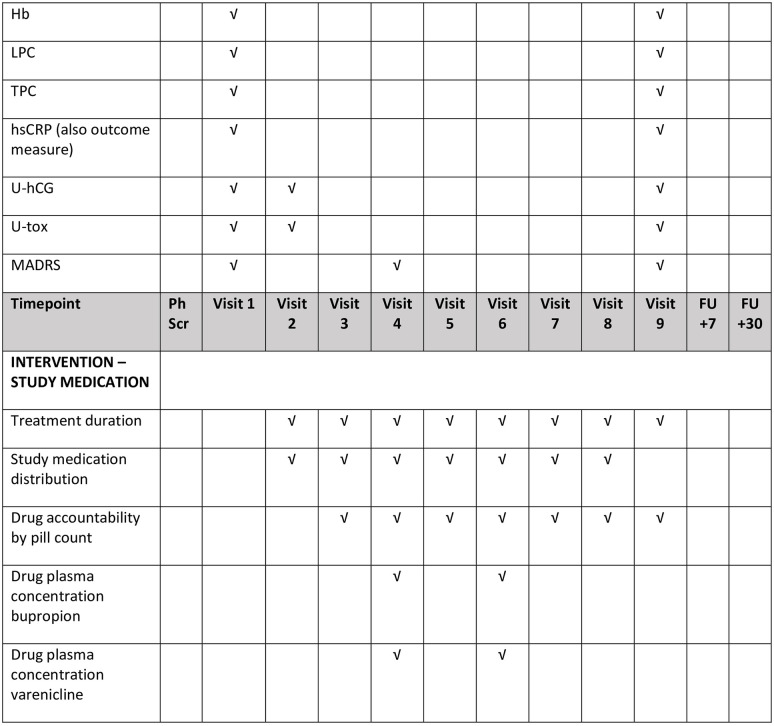
SPIRIT schedule of enrolment and summary of study assessments in the COMB study. AE = adverse event; ALT = alanine aminotransferase; ASRS = adult ADHD self-report scale; AST = aspartate aminotransferase; AUD = alcohol use disorder; AUDIT = alcohol use disorder identification test; B-PEth = blood phosphatidylethanol; CDT = carbohydrate deficient transferrin; CPTA = continuous performance test + activity; DUDIT = drug use disorder identification test; FU = follow up; GGT = gamma-glutamyltransferase; Hb = haemoglobin; hsCRP = high sensitive C-reactive protein; LPC = leukocyte plasma count; MADRS = Montgomery Åsberg depression rating scale; Na^+^/K^+^ = sodium/potassium; PC = prothrom bin complex; Ph = phone; QF = quantity frequency; SAE = serious adverse event; Scr = screening; TEPS = temporal experience of pleasure scale; TLFB = timeline follow-back; TPC = trombocyte particle concentration; U-hCG = urine human chorionic gonadotropin; U-tox = urine toxicology; VAS = visual analogue scale. *If judged as necessary by medical physician or study participant.

**Fig 2:**
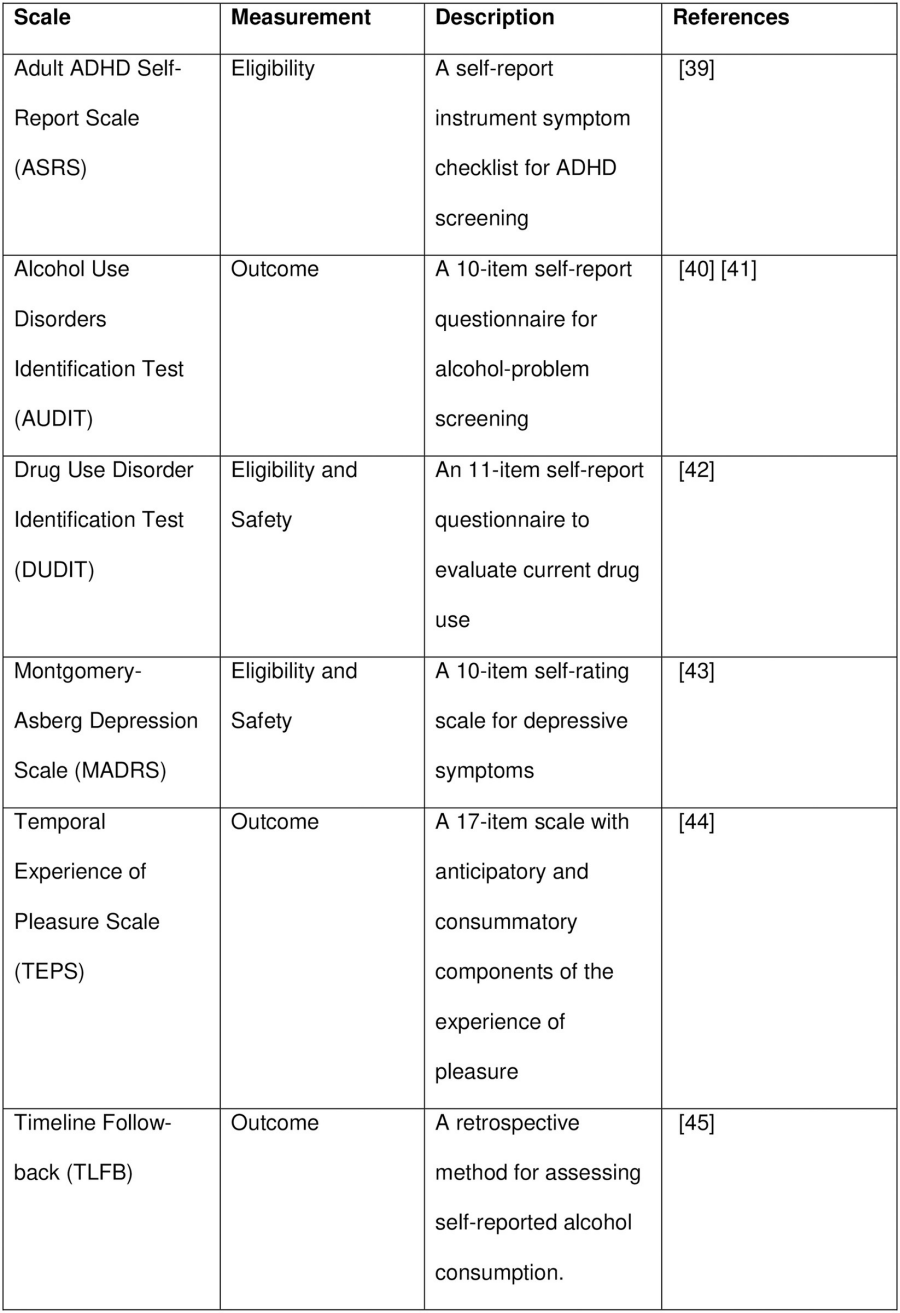
Scales and questionnaires used in the COMB study.

The following parameters will be measured/determined: breath alcohol concentration (BrAc) to ensure sobriety during both informed consent and cognitive testing (CPTA); demographics (gender, date of birth, civil status, number of children, and education level); alcohol-specific factors (answers to questions specifically related to quantity, frequency, family history of alcohol dependence, age of alcohol debut); nicotine-specific factors (answers to questions specifically related to quantity, frequency, age of debut); AUD diagnosis meeting ≥ 4 criteria according to DSM-V by structured interview; medical history (i.e., previous psychiatric and somatic disorders); routine physical examination, including heart and lung auscultation and palpation of abdomen; and, vital signs (blood pressure [systolic and diastolic] and heart rate after 10 minutes of rest, weight, and height). The study assessments and timelines are shown in Fig 1, and the scales and questionnaires are summarised in Fig 2.

#### Safety endpoints

The following parameters will be monitored as safety endpoints: levels of aspartate aminotransferase, alanine aminotransferase, prothrombin complex, creatinine glucose, Na^+^/K^+^, haemoglobin, the leukocyte plasma count, the thrombocyte particle concentration, high-sensitive C-reactive protein, human chorionic gonadotropin (for fertile women), urine toxicology, and the MADRS and DUDIT self-reporting scales for depression and drug abuse, respectively, according to the schedule shown in Fig 1.

### Study and participant timelines

**Fig 3:**
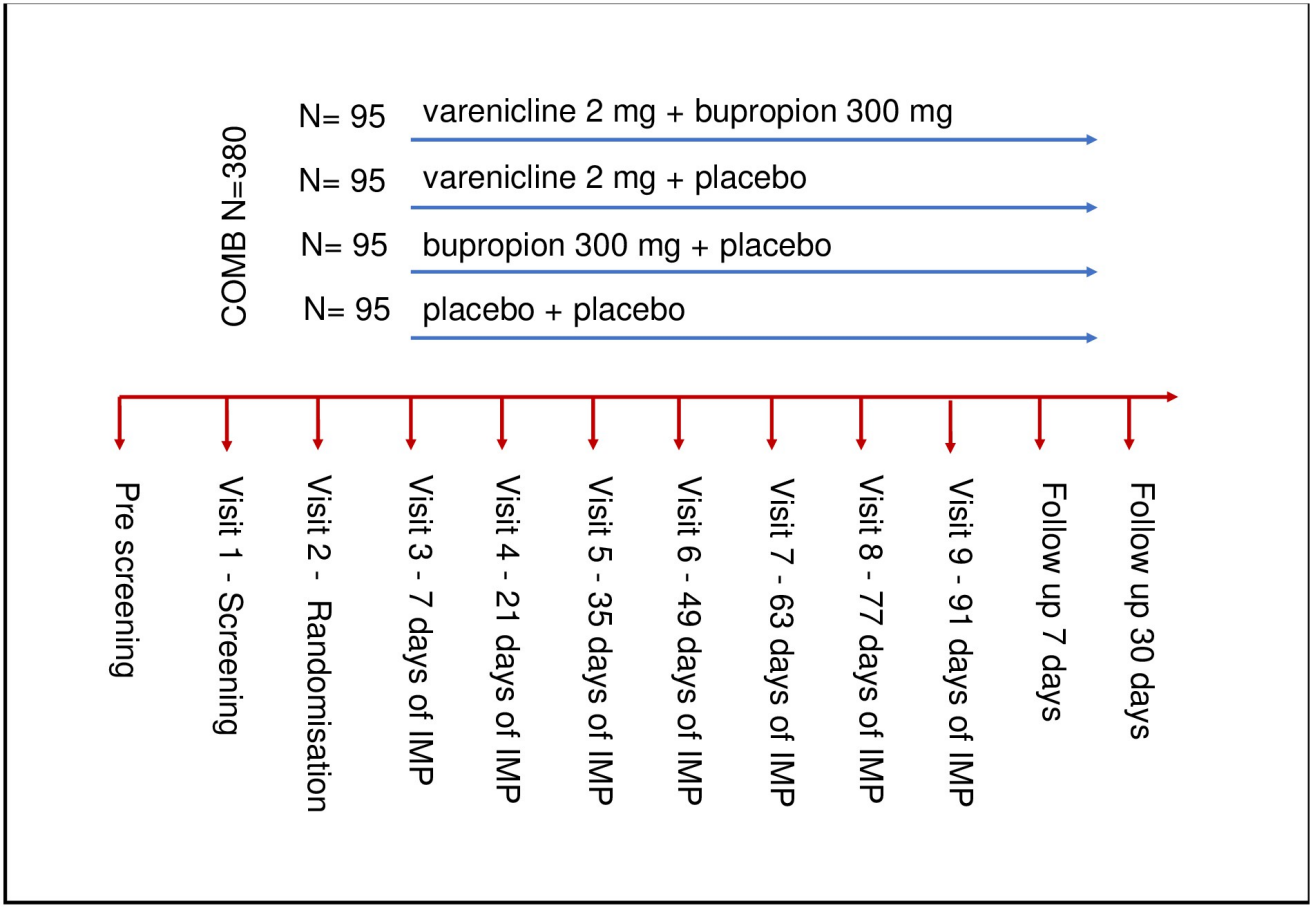
Treatment arms and schematic time line for study visits.

The timeline for the COMB Study is depicted in Fig 3. All study visits are summarized Fig 1. At all visits and follow ups, the study outcome measures HDD (according to TLFB) will be collected; all AEs will be documented and evaluated as regards any potential associations with the IMPs/study procedure and level of seriousness (potential SAEs and suspected unexpected serious adverse reactions will be evaluated and treated according to standard of care at the investigating centre); and, concomitant medications will be documented and any potential impact on safety and study results evaluated. At all visits, blood for B-PEth analysis will be collected and vital signs will be defined.

Potential participants who respond to media advertisements will be identified via pre-screening by phone. At Visit 1 (screening/baseline visit, acceptable range < +14 days from pre-screening), subjects’ breath alcohol levels will be evaluated, after which subjects must provide a signed, written Informed Consent Form before eligibility criteria will be evaluated; and, the study outcome variables (except for B-Peth) and safety parameters will also be evaluated. At Visit 2 (randomization visit, accepted range < +10 days from screening visit), eligibility criteria will be evaluated and subjects will receive a patient emergency card. Visits 3, 5 and 7 comprise the core battery visits (accepted range +/- 3 days from scheduled visit date). At Visit 4 (core battery, IMP concentration and safety visit, accepted range +/- 3 days from scheduled visit date), all safety parameters will be evaluated and blood samples taken to determine the IMP concentrations. At Visit 6 (core battery and IMP concentration, accepted range +/- 3 days from scheduled visit date), blood samples will be taken to determine IMP concentrations. At Visit 8 (end of analysis visit, accepted range +/- 3 days from scheduled visit date), study outcome variables and safety parameters will be evaluated. At Visit 9 (end of treatment/medical visit, accepted range +/- 3 days from scheduled visit date), subjects will undergo a medical examination by the physician. Subjects will be followed up by phone 7- and 30-days post end of treatment.

The COMB Study comprises a double-blind phase with nine visits in total over 13 weeks (91 days). Days 0‒7 indicate the titration of dosing.

### Sample size and recruitment

A total of 380 eligible subjects (95 subjects per treatment arm) will be recruited via adverts in newspapers, television, and social media for randomization into the study. To ensure adequate recruitment and compliance to study protocol, two advertising periods per year will be performed. Potential subjects will be evaluated in batches to maximize enrolment. Several different media will be used to maximize the reach of potential subjects of different genders and ages.

The power analysis for total N is based on two independent primary hypotheses: (i) B-PEth differs significantly between varenicline and bupropion alone and in combination vs. placebo, and (ii) the proportion of HDD differs significantly between varenicline and bupropion alone and in combination vs. placebo. With α = 0.025, 1-β = 0.80 and considering the outcome with the highest predicted sample size, total N has been estimated to be 360. The drop-out rate is estimated to be approx. 20% [24] between screening and randomization, and approx. 6.5% for randomization of subjects included in the modified ITT population. Thus, a sample size of approximately 380 subjects will be randomized.

### Randomization, allocation and blinding

Apotekets Production och Laboratorium, the appointed manufacturer of the IMPs, will package and label varenicline and placebo, and bupropion and placebo as two separate IMP kits. The manufacturer will use a blinding procedure (based on the internal standard operating procedure of the manufacturer), where the respective treatment kits for IMP 1 and IMP 2 will have been given a unique, sequentially numbered randomization number, generated randomly. Randomization will be performed according to block randomization (block-size 8). Each IMP will be allocated blinded to study sites. Subjects who meet the eligibility criteria will be randomly assigned to one of four treatment groups by the site study investigator in a 1:1:1:1 ratio to one of the following treatments: Varenicline + Placebo; Bupropion + Placebo; Varenicline + Bupropion; or Placebo + Placebo (Fig 3). Treatment will be blinded to subjects, investigators, and study personnel. Data will be analysed blinded by a statistical data analyst before the code is broken.

Opaque, sealed emergency envelopes containing information about each subject’s treatment will be kept at the Coordinating Centre.

### Data collection

All scales and questionnaires that will be used in the COMB Study to evaluate the eligibility and outcome measures (Fig 2) are validated and have high reliability [39–46]. Study personnel collecting trial data will be proficient in all study assessments and procedures. To enhance subject retention, all site visits will be planned at the randomisation visit and subjects will be contacted by phone at any deviation from the visit plan. All study personnel will be proficient in collecting and handling samples for blood, urine, and saliva testing. The laboratory tests are documented in “Measures” and Fig 1. Subjects who discontinue the study will be followed up by phone after 7 days and 30 days, according to protocol. Data collection forms will be written in Swedish (available upon request).

### Data management

All study-specific data will be recorded coded in the electronic Case Report File (eCRF), available only to delegated study personnel educated in data entry, password protected, and stored in secure servers. Data entry will be performed at each site in association with data collection. Data quality will be ensured by built in range and data validation checks. Data are audit trailed and monitored and signed by the investigator at each site.

Data management will be performed according to the Data Management Plan (S2 File). Sanity checks will be run on all included subjects, approximately once per month. A Data Manager will be responsible for database lock that will be performed after clean file has been declared and the Principal Investigator has downloaded an independent copy of the eCRF data for archiving.

### Statistical analysis

The mean values of each variable (primary and secondary) for the different treatment groups will be compared hierarchically using Analysis of Covariance (ANCOVA). Testing between treatments is performed hierarchically so no adjustment for multiple testing will be performed. Sensitivity to missing data will be evaluated by imputation methods, and robustness of results will be evaluated by sensitivity analyses as per the Statistical Analysis Plan (SAP; S3 File).

#### Exploratory analyses

Exploratory analyses will be performed on the impact of demographic data on the aforementioned primary and secondary endpoints; the relationship between the primary and secondary outcome measures; and, the effects of the primary and secondary outcome measures in relation to blood drug concentrations of bupropion and varenicline. Different models and *ad hoc* analyses will be performed, as specified in the SAP.

#### Subgroup analyses

Subgroup analyses will be performed on: gender; heredity (defined by family history positive); alcohol consumption levels at screening (defined by the World Health Organization consumption level categories: 0‒40 g/day, 40‒60 g/day, 60‒100 g/day and > 100 g/day); DSM diagnostic criteria for severe AUD at screening (defined by ≥ 6 criteria); early debut of alcohol consumption (age ≤ 12 years) as a proxy for early-onset AUD; responders/non-responders (defined by either a downward shift of two WHO consumption level categories measured by total alcohol consumption vs. baseline or a ≥ 50% reduction in B-PEth levels vs. baseline); early responders (defined by either a ≥ 50% reduction in B-PEth levels or a ≥ 50% reduction in HDD or a ≥ 50% reduction in total alcohol consumption between Visit 1 [screening] and Visit 2 [randomization/start IMP]). These analyses will be performed as per the SAP using different models and *ad hoc* analyses and divided by subgroups as defined.

#### Analysis sets

The modified ITT analysis set will include all study subjects who complete both the screening and randomization visits and report having taken at least one dose of the randomized study drugs, and they will be analysed according to their randomized study drug.

The Per Protocol analysis set will constitute all subjects from the ITT population considered to be “completers”. A completer is defined as a subject who has taken the IMP at least 80% of the planned number of days of the active treatment period and provided successful outcome measures of B-PEth and/or TLFB at screening and visits 2, 4, 6 and 8.

The safety analysis set will include all subjects who report having taken at least one dose of randomized study drug and for whom any post-dose data is available until Visit 8 (safety visit). Subjects will be analysed according to the actual treatment.

### Monitoring

#### Data monitoring and auditing

The study will be monitored and independently audited according to the monitoring plan (S4 File) by a monitor independent of the Sponsor and with no competing interests. All monitoring will be documented and trailed in the eCRF and the monitor will report to the Sponsor. An independent audit will be performed by CGP Consult. No interim analyses will be performed.

#### Harms

Adverse events will be coded using the Medical Dictionary for Regulatory Activities (MedDRA). All AEs that are observed by study personnel or reported by the subject during the trial will be recorded with the following information: description, date of onset and end date, severity, assessment of relatedness to trial medication and action taken, and whether or not it is attributable to trial medication. Adverse events will be assessed and authorized by a responsible medical physician. If considered to related to the trial medication, the AEs will be followed either until resolution or the event is considered to be stable. All SAEs will be reported on the SAE Reporting Form to the Sponsor or delegate within stipulated time frame. All Suspected Unexpected Serious Adverse Reactions (SUSARs) will be reported by the Sponsor to the relevant Competent Authority, the Ethics Committee, and all participating investigators, and reported via the Swedish Medical Products Agency to the Eudra Vigilance system within stipulated time frame.

### Ethics and dissemination

#### Research ethics approval, protocol amendments, and consent

The study will be performed in accordance with the study protocol, the WMA Declaration of Helsinki (October, 2013), GCP principles [i.e., ICH-GCP E6(R2)], all applicable regulatory requirements (i.e.,“Läkemedelsverkets föreskrifter”, LVFS 2011:9), and the EU General Data Protection Regulation (GDPR) 2016/679 privacy legislation. Ethical approval has been obtained from the Swedish Ethical Review Authority (EPM; D.nr. 431–18, 2018-06-18, amendment 2019-07-05/2019-11-26 D.nr. 2019–03559, amendment 2020-10-21 D.nr. 202004924, amendment 2022-01-14 D.nr 2021-06785-02). Approval has been obtained from the Swedish Medical Product Agency (MPA; EudraCT 2018–000048–24, amendment 2019-07-31 D.nr.5.1-2019-50751, amendment 2022-01-31 D.nr. 5.1-2021-101134). All subjects must provide written informed consent, obtained by delegated medical physician, to enter the study and for collection of all data relevant to the study, as per protocol. The full protocol and SAP, including any amendments, can be accessed in S3 and S5 Files, respectively.

#### Confidentiality

Participation in the COMB study will be recorded in a medical chart according to the Swedish Patient Data Act (2008:355) and informed consent will comply with relevant data protection, i.e., the EU General Data Protection Regulation (GDPR) 2016/679 privacy legislation. Study data will be stored in a computer database, maintaining confidentiality in accordance with national data legislation. The identities of the subjects will not be disclosed in any stored data.

#### Ancillary and post-trial care

Trial participants will be insured by the Swedish Patient Injury Act and the Swedish Pharmaceutical Insurance (Läkemedelsförsäkringen, see http://lff.se/).

#### Dissemination policy

Last patient out is estimated to occur in December, 2022 and study results will be submitted for publication in a scientific, peer-reviewed journal during 2023/2024. All authors will fulfil the ICMJE guidelines for authorship, and will also agree to be accountable for all aspects of the work.

## Discussion

In this study we have chosen varenicline and bupropion, two drugs with different but complimentary mechanisms of action to enhance dopamine levels in the brain, to challenge the low-dopamine hypothesis of alcohol addiction. By combining drugs with dopamine action, we seek to exceed the effect size of currently available treatments for AUD.

In AUD, human studies indicate that both pre- and post-synaptic aspects of basal dopamine neurotransmission are reduced [47, 48]. The resultant compromised dopamine system [49] may be genetically determined and/or develop as an adaptation to chronic alcohol use [50]. Furthermore, reduced dopamine neurotransmission has been associated with increased alcohol intake, as well as increased craving and alcohol-cue reactivity, both in animal [51, 52] and human [53] studies. The low-dopamine state is hypothesised to drive alcohol intake by negative reinforcement [18].

Trials that investigate the beneficial effects of enhancing dopamine neurotransmission are sparse, and studies that apply agents to increase endogenous dopamine levels are essentially lacking, except for those using varenicline. Moreover, the exact mechanism(s) underlying the observed effects of varenicline on AUD in RCTs [24, 25] have not been established. A reasonable hypothesis, however, is that they derive from its dopamine-elevating effect, and that the reported studies represent proof-of-concept that elevating central dopamine levels reduces alcohol intake.

We assume that further dopamine elevation is likely to enhance the effect and, indeed, recent evidence from animal studies that co-administration of varenicline and the dopamine-noradrenaline reuptake inhibitor bupropion enhances both the dopamine output in the nucleus accumbens and the reduction of the alcohol deprivation effect produced by varenicline supports this assumption [31].

The mechanisms by which varenicline and bupropion produce their limited dopamine elevations cannot be overridden by adding more drug of the respective drug, which limit the risk of abuse. Both drugs, however, are well tolerated and safe across various populations, and have been recommended in patients with psychiatric or addictive comorbidity [54].

In conclusion, the present study aims to challenge the low-dopamine hypothesis of AUD by pharmacologically mitigating the low-dopamine state that is thought to drive alcohol intake, whilst simultaneously evaluating the efficacy of the combination of varenicline and bupropion, which have complimentary mechanisms of action on the brain dopamine system, on alcohol consumption in AUD.

### Strengths

The COMB study is scientifically sound as regards power and design with biweekly data collection and an objective alcohol consumption blood marker (B-PEth) as one of the primary outcome measure. Moreover, B-PEth as an inclusion criterion will also ensure a high level of recent alcohol consumption. Additional design strengths include a sufficient time window between pre-screening, screening, and randomisation as this will reduce recruitment procedure discrepancies between sites, and recruiting potential trial participants in batches via advertisements to maintain the recruitment timeline. Since no psychotherapy will be administered, this will also strengthen the focus on the efficacy of the IMP under investigation.

Importantly, this study protocol will enable us to comprehensively evaluate the efficacy of varenicline and bupropion, either alone or in combination, in the AUD population. Alcohol is a major cause of premature death and disability and, with few available treatments that are of moderate efficacy, the need for new treatment options is pressing. Expanding our understanding of the mechanisms underlying alcohol dependence is also vital so in addition to the efficacy outcome, the secondary and explorative study outcomes will also shed light on the dopamine deficiency theory, enabling us to interrogate the dopamine hypothesis associated with addictive diseases. Notably, this study will be the first to use B-PEth for alcohol intake as a primary outcome variable in order to increase the chance of detecting treatment effects that may drive future study guidelines in AUD.

### Limitations

The COMB Study has been conducted during the Covid-19 Pandemic (2020‒21). Although local restrictions associated with the pandemic have influenced study procedures in some instances, our overall assessment is that the COMB Study has been performed according to protocol.

In July 2021, Pfizer globally withdrew varenicline (Champix^®^, Chantix^®^) from the market due to levels of N-nitroso-varenicline reported as being above acceptable limits. Since the IMP 1 for the COMB Study has been produced in batches due to sustainability factors, the final batch of IMP 1 produced in November 2021 contained the varenicline equivalent, Apo-Varenicline (Apotex^®^), with no reports to date of nitrosamine levels that would be expected to affect safety or study results. The last batch of IMP has also been subject to a limited shelf life due to distribution issues with varenicline, although this has had no negative impact on the study.

In summary, the COMB Study aims to be of value to the international AUD community not only by testing/validating a new pharmacological treatment strategy for AUD for which there is a high clinical need, but also in challenging the long-standing, low-dopamine hypothesis of addiction, which may lead to novel AUD treatment strategies.

## Supporting information

S1 File SPIRIT checklist for the COMB study. (PDF)

S2 File Data management plan for the COMB study. (PDF)

S3 File Statistical analysis plan for the COMB study. (PDF)

S4 File Monitoring plan for the COMB study. (PDF)

S5 File Protocol for the COMB study. (PDF)

S1 Table Sites participating in the COMB study. (PDF)
