# Supplementary material for: A randomized, double-blind, placebo-controlled, multicentre trial on the efficacy of varenicline and bupropion in combination and alone for treatment of alcohol use disorder: Protocol for the COMB study
Source: PLoS One. 2024 Jan 11;19(1):e0296118. doi: 10.1371/journal.pone.0296118 (PMC10783749; doi:10.1371/journal.pone.0296118)
Supplement: S2 File — (PDF) [file pone.0296118.s002.pdf]

Data Management Plan (DMP)

STUDY: COMB

Approval signatures:

____

Annika Wennersten, Data Manager

____

Niklas Svensson, DM Oversight Manager

____

Bo Söderpalm, Principal Investigator (Sponsor)

Transaction 09222115557481968755 Signed NS, AW, BS

Document Revision History

Version	Date	Author	Summary of Changes
v1.0	14 NOV -2022	Annika Wennersten	*N/A (First version)*

Transaction 09222115557481968755 Signed NS, AW, BS

Table of Contents

Document Revision History 2
Table of Contents 3
Acronyms and Abbreviations 4
1 Purpose 5
2 Key Roles 5
3 Reference Procedures 5
4 Study Design, Subjects and Sites 5
4.1 Study Design 5
4.2 Subjects 6
4.2.1 Planned Number of Subjects 6
4.2.2 Subject Identifiers 6
4.3 Sites 6
5 eCRF 6
5.1 eCRF System 6
5.2 eCRF Setup 6
5.3 eCRF Training 6
5.4 eCRF User Access 6
5.5 eCRF Data Validation Plan 7
6 Other (non-CRF) Study Data 7
6.1 Randomization 7
6.1.1 Description 7
6.1.2 Data Acquisition/Transfer Procedure 7
6.1.3 Data Validation Plan 7
6.2 CPTA 7
6.2.1 Description 7
6.2.2 Data Acquisition/Transfer Procedure 7
6.2.3 Data Validation Plan 8
6.3 PK Data 8
6.3.1 Description 8
6.3.2 Data Acquisition/Transfer Procedure 8
6.3.3 Data Validation Plan 8
6.4 Protocol Deviations 8
6.4.1 Description 8
6.4.2 Data Acquisition/Transfer Procedure 8
6.4.3 Data Validation Plan 8
7 Data Standards 8
8 Randomization 8
8.1 Misrandomizations 9
9 Blinding 9
10 Reports & Metrics 9
10.1 Query List 9
10.2 AE/SAE List 9
11 Data Safety Monitoring Board (DSMB) 9
12 Coding Dictionaries 9
13 Protocol Deviations 9
13.1 Data Checks to Identify Protocol Deviations 10
14 SAE Reconciliation 10
15 Interim Analyses 10
16 Statistical Analysis Preparations 10
17 Clean File and Database Lock 10
17.1 Preparations 10
17.2 Analysis Populations (only applicable for final Clean File) 11
17.3 Clean File Declaration 11
17.3.1 Clean File Meeting 11
17.4 Database Lock Procedures 11
17.5 Post Database Lock Procedures 11
17.5.1 Communication 11
17.5.2 Blinding/Un-blinding 11
17.5.3 Data Transfer 11
17.5.4 Principal Investigator Copy of Data 12

Transaction 09222115557481968755 Signed NS, AW, BS

18 Data Archiving/Retention **12**
APPENDIX A – Appointed DM Roles Log **13**
APPENDIX B – Data Cleaning Checks Specification **14**

Acronyms and Abbreviations

Abbreviations

AE	Adverse Event
APL	Apotek, Produktion och Laboratorier
BUP	Bupropion
CFM	Clean File Meeting
CPTA	Continuous Performance test + Activity
CRF	Case Report Form
CSP	Clinical Study Protocol
CSV	Comma Separated Values (a standard data file format)
DM	Data Management
DMP	Data Management Plan
DMR	Data Management Report
DM-TMF	Data Management Trial Master File
eCRF	Electronic Case Report Form
IMP	Investigational Medicinal Product
ISF	Investigator Site File
N/A	Not Applicable
PI	Principal Investigator
SAE	Serious Adverse Event
SAP	Statistical Analysis Plan
SAS	A software for data processing and statistics
SIV	Site Initiation Visit
SOP	Standard Operating Procedure
TDD	Technical Design Document
TMF	Trial Master File
VAR	Varenicline

Transaction 09222115557481968755 Signed NS, AW, BS

1 Purpose

The purpose of the Data Management Plan (DMP) is to define, within the scope of the study, the planned Data Management activities/timelines, data flows (inputs/processing/transfers/outputs), DM tools/systems and DM deliverables.

The DMP will be updated during the study if new information becomes relevant.

2 Key Roles

The following are key roles (either directly involved in the DM process, or with a significant interaction or dependency relationship):

- Data Manager, Medicase AB
- Clinical Trial Manager, Beroendekliniken SU
- eCRF Designer, Medicase AB
- Data Programmer, Medicase AB
- Medical Coder, Study sites
- Study Statistician, Statistiskkonsulterna
- DM Oversight Manager, Medicase AB

3 Reference Procedures

The Data Management will be performed under MediCase AB SOPs 900-999, at Data Management Service Level (DM-SL) 2 (refer to SOP 900).

SOP900 - Clinical Data Management

SOP910 - DM Trial Master File (DM-TMF)

SOP921 - eCRF Administration & Maintenance

SOP930 - Study Database

SOP940 - Data Cleaning & Validation

SOP950 - Data Management Programming

SOP962 - SAE Reconciliation

SOP990 - Clean File & Database Lock

SOP991 - Database Unlock

SOP995 - DM Project Closeout

***NOTE:** "SOP920 - eCRF Design, Setup, QC, Release & Training" and "SOP960 – Randomization" not applicable, eCRF already delivered before agreed (in Nov 2021) that MediCase will provide Data Management services.*

4 Study Design, Subjects and Sites

4.1 Study Design

Randomized, double-blind, placebo-controlled multicenter trial with four parallel groups.

Transaction 09222115557481968755 Signed NS, AW, BS

4.2 Subjects

4.2.1 Planned Number of Subjects

Planned number of screened subjects: Unknown (as many as needed to reach 391 randomized subjects).

Planned number of randomized subjects: 391 (equally divided per treatment arm)

4.2.2 Subject Identifiers

Screened subjects will automatically be assigned a Screening ID number when added to the eCRF system. The Screening ID is on the format S-9XXX, where S is the site number and XXX is a sequential number within the site.

Upon randomization, the subjects will receive a Subject ID number equal to the randomization number in the randomization list. The Randomization Number/Subject ID is on the format XXX, where XXX is a number starting at 001. After randomization the Subject ID number will be the primary ID used for the subject.

ID numbers (neither Screening ID nor Randomization Number/Subject ID) will never be reused (in case of subject discontinuation or for any other reason).

4.3 Sites

Site #	Name	Location	Notes
1	Beroendekliniken SU, Sahlgrenska University Hospital	Gothenburg, Sweden	
2	Linköping University Hospital	Linköping, Sweden	
3	Beroendecentrum Stockholm	Stockholm, Sweden	
4	Psykiatriforskning Skåne/Beroendecentrum Malmö	Lund, Sweden	

5 eCRF

5.1 eCRF System

MediCase eCRF, provided by MediCase AB, Gothenburg, Sweden. It is a validated eCRF system. The database is located in Sweden. System documentation maintained by MediCase AB.

5.2 eCRF Setup

eCRF has been set up, based on the study protocol, forms/questionnaires, and other specifications supplied by the Sponsor. The Clinical Trial Manager (Sponsor) approved release to production. Any changes to the eCRF after the effective date of v1.0 of this Data Management Plan will be done according to SOP921 - eCRF Administration & Maintenance.

5.3 eCRF Training

MediCase provided training to the Sponsor, who instructs the Investigator and site personnel in the use of the eCRF. MediCase provides user support to the Sponsor as needed.

5.4 eCRF User Access

Clinical Trial Manager sets up (or edit/remove if applicable) user accounts. MediCase provides user account administration support upon request by the Clinical Trial Manager.

Transaction 09222115557481968755 Signed NS, AW, BS

List of relevant eCRF user roles:

User Role	Description of privileges
Data Entry User	May enter data at the local site
Local Investigator	May enter data at the local site, and electronically sign those data
Data Manager	May raise queries, download data, and lock data
Study Designer	May set up and configure the study eCRF
Monitor	May raise queries and mark data as monitored
Sponsor	May read data
Study User Manager	May create new users, and assign role, at study scope

5.5 eCRF Data Validation Plan

The eCRF will have built-in data validation checks, see eCRF Technical Design Document (TDD) for a list.

For data cleaning checks performed outside the eCRF, see the eCRF data domain in APPENDIX B – Data Cleaning Checks Specification.

6 Other (non-CRF) Study Data

The complete study database consists, in addition to the eCRF data, of the following data classes/domains, each further described in sub-chapters below:

Data Class	Data Source	Notes
Randomization	Study Statistician	Unblinding data
CPTA	Opatus	Opatus CPTA Attention Assessment App.
PK Data	Q&Q Labs	Steady State serum concentrations of varenicline and bupropion
Protocol Deviations	Sponsor	Classification of protocol deviations

6.1 Randomization

See section *8 Randomization* for additional related information.

6.1.1 Description

See section *8 Randomization* for a description of the randomisation procedure.

6.1.2 Data Acquisition/Transfer Procedure

After clean file and database lock, the Randomization List will be provided to Data Management by unblinded Statistician, to be added to the study database.

6.1.3 Data Validation Plan

N/A

6.2 CPTA

6.2.1 Description

CPTA is a neuropsychiatric tool that addresses the three entities inattention, impulsivity, and activity. The test is administrated via a mobile unit (iPod) and the test time is 20 minutes/timepoint. Opatus CPTA is used in the trial as a proxy to assess the hypofrontality/hypodopaminergic (frontal cortex) hypothesis of AUD.

6.2.2 Data Acquisition/Transfer Procedure

The data will be delivered by courier or by an Opatus representative in person by means of data in csv-format on a USB stick. The data will be encrypted and protected by a password. An off-line backup copy of the data will be saved at Opatus premises until asked to delete by the project.

Transaction 09222115557481968755 Signed NS, AW, BS

6.2.3 Data Validation Plan

Reconciliation to verify that when stated in eCRF that CPTA was performed there is a date matched record in the CPTA data file.

6.3 PK Data

6.3.1 Description

No PK data files may be sent from the lab until after the rest of the study database is locked.

6.3.2 Data Acquisition/Transfer Procedure

Data will be transferred, from Q&Q LABS, as a single excel file via email to a sponsor-dedicated person. The file will be Zip file and password protected. The file name will be COMB-B08_YYYYMMDD.zip. The sponsor will produce a list, containing appropriate variables, for Data Management to be able to run a reconciliation between PK data and the eCRF.

6.3.3 Data Validation Plan

Reconciliation to verify that when stated in eCRF that PK sample was taken there is a date matched record in the PK data file.

6.4 Protocol Deviations

6.4.1 Description

A List of protocol deviations entered in eCRF, with classifications made by Sponsor.

6.4.2 Data Acquisition/Transfer Procedure

A custom report with all protocol deviations entered in the eCRF will be extracted, as an excel-file, by the Sponsor who will then add appropriate classifications and send the file back to Data Management as a password protected file.

6.4.3 Data Validation Plan

Reconciliation to verify that protocol deviations entered in eCRF match the records in the file from the Sponsor.

7 Data Standards

N/A

8 Randomization

APL, appointed manufacturer of the IMPs, have packed and labelled, VAR and placebo and BUP and placebo, respectively, in two separate IMP kits. APL have used a blinding procedure according to internal standard operating procedure, where two associated treatment kits, one for IMP 1 and one for IMP 2 have been given a unique number (a Randomization number) generated randomly.

Subjects have been randomized strictly sequentially, in a 1:1:1:1 ratio to one of the following treatments:
1) Varenicline + Bupropion SR
2) Varenicline + Placebo
3) Bupropion SR + Placebo
4) Placebo + Placebo
Each randomized subject have been allocated a Randomization number, chronologically, according to the Randomization list. The Randomization number defines the IMP1 and IMP 2 and is therefore equal for series 1 and series 2.

Transaction 09222115557481968755 Signed NS, AW, BS

8.1 Misrandomizations

Misrandomizations are cases where randomization was made by the site either prematurely or inappropriately prior to confirmation of the subject's final randomization eligibility. Mistakenly randomizing a subject who does not meet the inclusion/exclusion criteria will be considered a protocol violation. For more information, please see section 6.5 in the SAP.

9 Blinding

Treatment is double blinded, i.e., neither study personnel, nor study participants will know if they are given active study product or placebo. Sealed emergency envelopes, containing information about each subject's treatment, will be kept by the Sponsor, in a locked cabinet, at the Coordinating center. In case of a medical emergency the Investigators at respective study center will call the 24 hours/day emergency telephone number to the Sponsor/Coordinating Investigator, who will open the envelope and directly communicate the subject's treatment by telephone to the Investigator.

10 Reports & Metrics

10.1 Query List

A query list, including resolution status, can be found in the eCRF, and can be viewed by all users.

10.2 AE/SAE List

An AE list, including if SAE or not, can be found in the eCRF, and can be viewed by all users. Each user will only see AEs for subjects at the site/sites the user has access to.

11 Data Safety Monitoring Board (DSMB)

N/A

12 Coding Dictionaries

The following items will be coded:

Item to be coded	Dictionary	Version	Coding process
Medical History Diagnosis	ICD10-SE	N/A	Coded in eCRF by site staff at time of data entry
Adverse Event	MedDRA	20.1	Coded in eCRF by site staff at time of data entry. Sponsor will review and approve all coding
Concomitant Medication	ATC	N/A	Coded in eCRF by site staff at time of data entry

13 Protocol Deviations

Protocol deviations will be recorded in the eCRF by site staff. Classifications of the protocol deviations will be made by the Sponsor *(see section 6.4.2)*. A list of important protocol deviations, including classifications, will be finalized prior to study un-blinding.

Transaction 09222115557481968755 Signed NS, AW, BS

13.1 Data Checks to Identify Protocol Deviations

Monitor will check for general site procedural protocol deviations during monitoring visits (findings will be recorded in the eCRF as described above).

Data Manager will apply checks identified as "Is Protocol Deviation" =Y in APPENDIX B – Data Cleaning Checks Specification.

14 SAE Reconciliation

The Sponsor will reconcile SAE details between the eCRF and source document(s). Any discrepancies found will be reviewed and addressed by the Sponsor.

The reconciliation will be performed before final clean file.

15 Interim Analyses

N/A

16 Statistical Analysis Preparations

See chapter *17 Clean File and Database Lock*. Any other special preparations (if needed) must be determined and agreed during the clean file process.

17 Clean File and Database Lock

17.1 Preparations

The following will be prepared before Clean File:

- Data should be entered into eCRF, and any queries should be resolved.
- CPTA results acquired *(see 6.2 CPTA*).
- Notify PK lab that a PK data file will soon be requested but note that the file may NOT be sent until after clean file declaration and database lock.
- Data Manager should complete data cleaning (according to DMP) and summarize in a *Data Management Report (DMR)* (appendix A to SOP 990).
- Sponsor initiates SAE reconciliation (see *14 SAE Reconciliation*).
- Medical coding should be finalized. Medical coding should be approved by Sponsor appointee.
- Monitoring should be completed according to Monitoring Manual.
- Statistical Analysis Plan (SAP) should be finalized and approved.
- Review of any Protocol Deviations (see *13 Protocol Deviations*).
- eCRF should be signed by PI.
- Review of if any emergency envelopes (see *9 Blinding*) have been used.
- Blind Data Review should be completed, where missing data and data quality will be assessed. Decisions based on the review should be taken *(see SAP Section 8.2)*.

Transaction 09222115557481968755 Signed NS, AW, BS

17.2 Analysis Populations (only applicable for final Clean File)

Three analysis sets will be used (Modified Intention-to-treat (mITT), Safety and Per-Protocol (PP)). These analysis sets are defined in section 8. STATISTICS in the study protocol and section 6 ANALYSIS SETS in the SAP.
The PP analysis set constitutes all subjects from the mITT population who are considered to be completers. A completer is defined as a subject who has taken the IMP at least 80% of the planned number of days of the treatment period. For a complete definition of the PP population, see section 6.3 Per-protocol (PP) analysis set in the SAP.

17.3 Clean File Declaration

17.3.1 Clean File Meeting

The purpose of the Clean File Meeting (CFM) is to assess the readiness of the database (e.g., that all data are considered cleaned, all non-CRF data are included and that there are no outstanding issues) and eventually decide if the database is to be locked or if further activities are needed before clean file can be declared. A series of meetings and other communication may be needed to eventually come to **the** clean file decision.

During the meeting the topics in sections *17.1 Preparations* and *17.2 Analysis Populations* will be checked to make sure nothing is missed or unresolved.

The Data Manager is responsible for coordinating and hosting the CFM activities. The CFM will be held by representatives from the study team. The Clinical Trial Manager (Sponsor), Data Manager, DM Oversight Manager and Study Statistician will attend the meeting. Monitor, and others may attend as relevant.

The Data Manager will write meeting minutes and a Clean File Report. The report will be finalized after the CFM and sent for approval by Clinical Trial Manager (Sponsor).

17.4 Database Lock Procedures

When clean file has been declared and documented with signatures, the Data Manager is responsible for locking the database by removing all write/edit access to the eCRF (only Data Manager will be able to un-lock the data). All non-CRF data (see *6 Other (Non-CRF) Study Data*) will be stored in DM-TMF and password protected.

Data Manager is responsible for documenting the database lock on a *Database Lock Form* (Appendix B to SOP 990).

17.5 Post Database Lock Procedures

17.5.1 Communication

Clinical Trial Manager will communicate to other relevant parties that the database has been locked.

17.5.2 Blinding/Un-blinding

The treatment/randomisation code must not be broken before the database has been locked.

When all clean file documentation is finalized and signed, the Data Manager will notify the study Clinical Trial Manager who may then request the Randomization List from the unblinded Statistician. The list may at this point be freely distributed as needed to other parties. PK data files should also be collected from the lab at this point.

17.5.3 Data Transfer

Study data (i.e., eCRF data and non-CRF data) will be sent by the Data Manager to the Study Statistician for further analysis.

eCRF data will be sent in SAS or CSV format and with variable specification list and annotated CRF. CGM data format to be determined later.

Study data will be sent either in a secure file upload function provided by the Sponsor or the Study Statistician, or in a file with AES password encryption using the software 7zip via email.

Transaction 09222115557481968755 Signed NS, AW, BS

17.5.4 Principal Investigator Copy of Data

After database lock, the PI must download an independent copy of the eCRF data, with audit trail and information regarding monitoring and PI signature shown, in "human readable format" (PDF file format) to be filed in the Investigator Site File (ISF). The PI must confirm successful download by email to either Site Monitor, Clinical Trial Manager, or Data Manager.

18 Data Archiving/Retention

The Data Manager is responsible for the DM-TMF. The Data Manager is responsible for transferring the final eCRF data (raw data in CSV format, including associated dataset/variable specification and annotated CRF, and "human readable format" PDFs) and final non-eCRF (data files) to the DM-TMF.

The DM-TMF (including the study data) will be transferred to the Sponsor, to be included in the study TMF.

The PI is responsible for archiving an independent copy of the eCRF data (see *17.5.4 Principal Investigator Copy of Data*).

Transaction 09222115557481968755 Signed NS, AW, BS

APPENDIX A – Appointed DM Roles Log

Role	Name	Start date	End date	Comment
Data Manager	Annika Wennersten	2021-11-08		
DM Oversight Manager	Niklas Svensson	2021-11-08		
eCRF Designer	Niklas Svensson	Start of Study		

Transaction 09222115557481968755 Signed NS, AW, BS

APPENDIX B – Data Cleaning Checks Specification

This Appendix lists data cleaning checks performed outside of the eCRF.

These checks will be run on included subjects, approximately once a month. The frequency may be adjusted, if needed, depending on the data quality.

For data validation checks built into the eCRF, see the eCRF Technical Design Document (TDD).

Data Domain	Check ID	Check Description	Is Protocol Deviation	Action
eCRF	IC01	Informed consent:<br><br>- Check that Informed consent has been signed no later than date of Visit 1. This check will be run on screening failures as well.	Y	Verify that recorded in the Protocol Deviations section of the eCRF, otherwise query in eCRF
eCRF	PE01	Check for any cases of Clinically Significant Physical Examination (Somatic Status) finding after Visit 1 are either present also at Visit 1 or reported as an Adverse Event	N	Query in eCRF
eCRF	LB01	Check for any cases of Clinically Significant lab abnormalities after Visit 1 are either present also at Visit 1 or reported as an Adverse Event	N	Query in eCRF
eCRF	LB02	Check for any cases of Clinically Significant lab abnormalities at Visit 1 are present also at Medical History	N	Query in eCRF
eCRF	LB03	Sanity checks on GGT and CDT (lab results) with focus on extreme values (will be run when needed, not on routine).	N	List output for Sponsor review
eCRF	BP01	Sanity checks on B-Peth results with focus on extreme values (will be run when needed, not on routine).	N	List output for Sponsor review
eCRF	HDD01	Sanity checks on HDD (TLFB results) with focus on extreme values (will be run when needed, not on routine).	N	List output for Sponsor review
CPTA data file	CPTA01	Check that if CPTA is performed according to eCRF, there is one row for the corresponding subject and visit-date in the CPTA datafile (will be run after received datafile).	N	Reconciliation – check CPTA datafile against eCRF
PK data file	PK01	Check that if PK sample performed according to eCRF, there is one row for the corresponding subject and visit-date in the PK datafile (will be run once, after received datafile).	N	Reconciliation – check PK datafile against eCRF
eCRF	MS01	Check that MADRS score >19 is reported as an AE.	N	Query in eCRF

Transaction 09222115557481968755 Signed NS, AW, BS

Verification

Transaction 09222115557481968755

Document

DMP COMB v1.0
Main document
14 pages
Initiated on 2022-11-22 14:48:48 CET (+0100) by Niklas Svensson (NS)
Finalised on 2022-11-27 19:31:20 CET (+0100)

Signing parties

Niklas Svensson (NS)
MediCase AB
ID number 7401244913
Company reg. no. 556967-8351
niklas.svensson@medicase.se
+46733611117

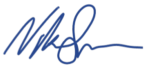

Signed 2022-11-22 14:50:42 CET (+0100)

Annika Wennersten (AW)
MediCase AB
annika.wennersten@medicase.se

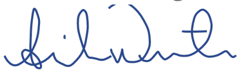

Signed 2022-11-25 11:06:39 CET (+0100)

Bo Söderpalm (BS)
Göteborgs Universitet
bo.soderpalm@neuro.gu.se

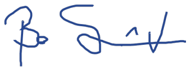

Signed 2022-11-27 19:31:20 CET (+0100)

This verification was issued by Scrive. Information in italics has been safely verified by Scrive. For more information/evidence about this document see the concealed attachments. Use a PDF-reader such as Adobe Reader that can show concealed attachments to view the attachments. Please observe that if the document is printed, the integrity of such printed copy cannot be verified as per the below and that a basic print-out lacks the contents of the concealed attachments. The digital signature (electronic seal) ensures that the integrity of this document, including the concealed attachments, can be proven mathematically and independently of Scrive. For your convenience Scrive also provides a service that enables you to automatically verify the document's integrity at: https://scrive.com/verify
