# Supplementary material for: A randomized, double-blind, placebo-controlled, multicentre trial on the efficacy of varenicline and bupropion in combination and alone for treatment of alcohol use disorder: Protocol for the COMB study
Source: PLoS One. 2024 Jan 11;19(1):e0296118. doi: 10.1371/journal.pone.0296118 (PMC10783749; doi:10.1371/journal.pone.0296118)
Supplement: S3 File — (PDF) [file pone.0296118.s003.pdf]

Statistikkonsulterna
JOSTAT & MR SAMPLE AB

Statistical analysis plan

A randomized, double-blind, placebo controlled, double-dummy, four-armed parallel-group, multicenter study during 12-weeks to evaluate/comparing the effect of varenicline and bupropion in combination and alone for treatment of alcohol use disorder.

**Date:**	**Prepared:**
2022-11-15	Magnus Pettersson
**Status:**	
Final	
**Change history**	
2019-10-29	Final version
2022-11-15	Updated PP criteria
**Signatures:** 2022-11-15	
**Magnus Pettersson, MSc**<br>Biostatistician, PStat®<br>Statistikkonsulterna	

Transaktion 09222115557481461024 Signerat MP

1 Innehåll

1 Innehåll 2

2 List of abbreviations 3

3 INTRODUCTION 4

4 OBJECTIVES 4
4.1 Primary objective 4
4.2 Secondary objectives 4

5 STUDY DETAILS 5
5.1 Study design 5
5.2 Sample size 5
5.3 Drop-out rate 5

6 ANALYSIS SETS 6
6.1 Definition of analysis sets 6
6.2 Intention-to-treat (ITT) analysis set 6
6.3 Per-protocol (PP) analysis set 6
6.4 Safety set 6
6.5 Violations and deviations 7
6.5.1 Compliance 7
6.5.2 Randomization issues 7

7 PRIMARY AND SECONDARY VARIABLES 7
7.1 Primary efficacy variable 7
7.1.1 PEth 7
7.1.2 Heavy Drinking Days (HDD) 7
7.2 Secondary efficacy variables 8

8 STATISTICAL AND ANALYTICAL PLANS 8
8.1 General principles 8
8.1.1 Visit windows 9
8.1.2 Baseline 9
8.1.3 Treatment period 9
8.1.4 Follow-up Period 9
8.1.5 Multiplicity 9
8.1.6 Handling of missing data 9
8.1.7 Handling of outliers 10
8.1.8 Patient disposition 11
8.1.9 Protocol Deviations 11
8.2 Assessment of data quality prior to clean file 11
8.3 Analysis of primary efficacy variable 11
8.3.1 PEth 12
8.3.2 Heavy Drinking Days (HDD) 13
8.4 Analysis of secondary efficacy variable 14
8.4.1 Additional alcohol markers 14
8.4.2 Subjective alcohol consumption 14
8.4.3 AUDIT 14
8.4.4 Alcohol craving (VAS) 14
8.4.5 Nicotine use 14

© Statistikkonsulterna

Transaktion 09222115557481461024 Signerat MP

8.4.6 Temporal experience of pleasure scale (TEPS) 15
8.4.7 Opatus®CPTA 15
8.4.8 Interaction of alcohol and nicotine consumption 15
8.5 Subgroup for exploratory analysis 15
8.6 Sensitivity analysis 16
8.7 Summary of efficacy analysis 16
9 INTERIM ANALYSIS 17
10 CHANGES OF ANALYSIS FROM PROTOCOL 17
11 REPORTING AND PUBLICATION OF RESULTS 17

2 List of abbreviations

Abbreviation	
SAP	Statistical Analytical Plan
CSP	Clinical Study Protocol
PEth	Phosphatidylethanol
HDD	Heavy Drinking Days
EoT	End of Treatment
ADHD	Attention Deficit Hyperactivity Disorder
AUDIT	Alcohol Use Disorder Identification Test
DUDIT	Drug Use Disorder Identification Test
TLFB	Time Line Follow Back
ITT	Intention to Treat
PP	Per Protocol
IMP	Investigational Medicinal Product
CDT	Carbohydrate Deficient Transferrin
GGT	Gamma Glytamyltransferase
U-EtG	Urine Ethyl Glucoronide
VAS	Vislual Analogue Scale
SD	Standard Deviation
FU	Follow up
FHP	Family History Positive
LOCF	Last Observation Carried Forward
MAR	Missing At Random
MNAR	Missing Not At Random
NA	Not Applicable

Transaktion 09222115557481461024 Signerat MP

3 INTRODUCTION

This statistical analysis plan (SAP) describes the (final) analyses to be performed at the account of Addiction Biology Unit. The SAP is based on the Clinical Study Protocol (CSP) Version 1, Lidö & deBejczy, 2018-02-09, with title "A randomized, double-blind, placebo-controlled multicenter trial on the efficacy of varenicline and bupropion, in combination and alone, for treatment of alcohol use disorder" and EudraCT No 2018-000048-24.

4 OBJECTIVES

4.1 Primary objective

To assess the effect and interaction of the smoking-cessation drugs varenicline and bupropion in combination and alone versus placebo for reducing alcohol consumption in individuals with alcohol use disorder (AUD).

Two primary efficacy endpoints will be used

1) Alcohol consumption as measured by the objective alcohol marker B-PEth
2) Alcohol consumption as measured by number of heavy drinking days (HDD) by using the self-reported Time Line Follow Back (TLFB) procedure.

4.2 Secondary objectives

1) To assess the effect and interaction of the smoking-cessation drugs varenicline and bupropion in combination and alone versus placebo for reducing alcohol consumption in individuals with AUD by using additional biological markers
   - carbohydrate deficient transferrin (CDT)
   - gamma glutamyltransferase (GGT)

2) To assess the effect and interaction of the smoking-cessation drugs varenicline and bupropion in combination and alone versus placebo for reducing alcohol consumption in individuals with AUD by using self-reported alcohol consumption measured by TimeLine Follow Back (TLFB):
   - Mean grams alcohol per day
   - Number of drinking days
   - Number of abstinent days
   - Number of drinks per drinking day

3 To assess the effects of the smoking-cessation drugs varenicline and bupropion in combination and alone versus placebo, on other clinically relevant efficacy endpoints:
   - Total score of Alcohol Use Identification Test (AUDIT)
   - Alcohol craving measured by Visual analogue scale (VAS)

Transaktion 09222115557481461024 Signerat MP

  - Nicotine use measured by nicotine saliva marker cotinine
  - Temporal experience of pleasure scale (TEPS)
  - The continuous performance test + Activity test (CPTA)

- To assess the relationships between the above described outcome measures and plasma drug concentrations of BUP and VAR measured at visit 4 and 6

5 STUDY DETAILS

5.1 Study design

Study design is described in detail in the CSP (version 1, 2018-02-09).

The study is designed as a Phase IIB, randomized, double-blind, placebo-controlled multicenter trial with 4 parallel groups, evaluating the effects of varenicline (drug A) and bupropion (drug B) in combination and alone compared to placebo for treatment of alcohol use disorder (AUD). (Arm 1: drug A+drug B, Arm 2: drug A+placebo, Arm 3: drug B+placebo, Arm 4: placebo + placebo). The treatment effect will be measured by change in alcohol consumption and the difference between treatment groups.

Duration: 13 weeks

5.2 Sample size

The power analysis is based on two independent primary hypotheses;

- PEth differ significantly between varenicline and bupropion in combination and alone versus placebo
- Proportion of heavy drinking days differ significantly between varenicline and bupropion in combination and alone versus placebo

With regards to PEth, we assume that we can use an ordinary t-tests based on normal distribution, with α=0.025, 1-β=0.80. and using a standard deviation of 0.8 (previous results from Varenicline study ref). Recruiting 320 patients, randomized in 4 groups of 80 each, we get a detectable effect of 0.35 umol/l and a Cohen's d of 0.44.

With regards to HDD, we assume that we can use ordinary t-tests based on normal distribution, with α=0.025, 1-β=0.80, using a standard deviation of maximum 0.35 (previous results from Varenicline study Litten ref). Recruiting 360 patients, randomized in 4 groups of 90 each, we get a detectable effect of 15% and a Cohen's d of 0.43.

Hence, a sample size of 360 patients should be randomized.

5.3 Drop-out rate

Based on prior studies (Varenicline for treatment of alcohol dependence, A de Bejczy, ACER 39(11):2189-2199, 2014) with similar design and outcome, the drop-out rate is estimated to approx. 20 % between screening and randomization and approx. 6.5% for

Transaktion 09222115557481461024 Signerat MP

randomization to subjects included in ITT. To balance the drop-out 380 subjects are estimated for screening and 360 subjects for randomization.

6 ANALYSIS SETS

6.1 Definition of analysis sets

Three analysis sets will be used: modified Intention-To-Treat m(ITT), Per Protocol (PP) and Safety. The primary and secondary efficacy endpoints will be estimated using mITT and PP analysis sets separately. The safety endpoints will be analyzed on the safety analysis set.

6.2 Modified Intention-to-treat (mITT) analysis set

The modified ITT analysis set will include all trial subjects who complete screening visit, and randomization visit and report that they have taken at least one dose of randomized investigational medicinal product (IMP). Subjects will be analyzed according to their randomized IMP.

6.3 Per-protocol (PP) analysis set

The PP analysis set constitutes all subjects from the mITT population who are considered to be completers. A completer is defined as a subject who:

Has taken the IMP at least 80% of the planned number of days of the treatment period Day 1 (day after visit 2, randomization) to Day 91 (visit 9).

Also, for the primary outcome PEth

Fulfill the milestones, where the milestones are met by subjects who have:

- successful PEth analyses at screening, Day 0, Day, 21, Day 49 and Day 77

for the primary outcome HDD

Fulfill the milestones, where the milestones are met by subjects who have:

- successful TLFB procedure at screening, Day 0, Day, 21, Day 49 and Day 77

In case of violation of any of the above-mentioned criteria the subject will not be included in the PP analysis set. Subjects will be analyzed according to their randomized study drug.

The PP population is divided into two sets due to the special circumstances of the Covid-19 pandemic 2020-2021, where site visits were sometimes inhibited and therefore PEth sampling. However, TLFB was still collected by phone.

6.4 Safety set

The safety analysis set includes all subjects who report that they have taken at least one dose of randomized study drug, and for whom any post-dose data are available up until

Transaktion 09222115557481461024 Signerat MP

Day 77.
Patients will be analyzed according to the actual treatment.

6.5 Violations and deviations

Violations and deviations will be described in the CSP (version 1, Lidö & debejczy, 2018-02-09).

6.5.1 Compliance

Adherence to the study drug regimen will be analyzed by pill count and by blood levels of drug/placebo markers.

6.5.2 Randomization issues

Mistakenly randomizing a patient who does not meet the inclusion/exclusion criteria will be considered a protocol violation. Any such patients will not be included in the analysis sets.

7 PRIMARY AND SECONDARY VARIABLES

7.1 Primary efficacy variable

7.1.1 PEth

One of the primary outcomes will be the levels of the objective alcohol marker Phosphatidylethanol (PEth) measured in blood.

The primary efficacy variable, PEth, is calculated as the mean value over the 8-week steady state active treatment period visit 4-visit 8 compared to baseline. The data is analyzed as the within patient difference.

Visit 9 (data from week 12 and 13) is omitted in the analysis period due to the tendency of drop-out and non-compliance at the end of a study period.
Additionally, PEth-level analysis compared to baseline levels will also be performed for visit 3, visit 4, visit 5, visit 6, visit 7, visit 8 and visit 9.

PEth has a well-documented [see CSP, Version 1, 2018-02-09) declining pattern and hence it follows that the mean value during the treatment period will be a sufficient statistic for the post-treatment level.

PEth will be collected initially at the screening visit and onwards from randomization visit every 2nd week during the study (except for the first visit, which is one week after randomization).

7.1.2 Heavy Drinking Days (HDD)

The additional main outcome will be alcohol consumption as measured by Heavy Drinking Days (HDD) using the Time Line Follow Back (TLFB) procedure.

The number of heavy drinking days HDD by 14 days is defined as a mean over the 8-week steady state active treatment period (visit 4- visit 8) compared to baseline.

Transaktion 09222115557481461024 Signerat MP

A HDD is defined as ≥5 standard drinks per day for men and ≥4 standard drinks per day for women. The baseline mean of heavy drinking days by 14 days, is obtained at the screening visit, where the number of heavy drinking days over the last 30 days is registered.

The number of heavy drinking days, "HDD visit 4- visit 8" = total number of heavy drinking days divided by 4 (8weeks/4), in order to get a 14-day-period measurements. The maximum total number of days measured is 56 days.

Additionally, HDD analysis compared to baseline levels will also be performed for visit 3, visit 4, visit 5, visit 6, visit 7, visit 8 and visit 9 and parts of the 8 week steady state active treatment period.

7.2 Secondary efficacy variables

Secondary outcomes will include;

- Additional alcohol markers defined as carbohydrate deficient transferrin (CDT) and Gamma glutamyltransferase (GGT) measured in blood
- Self-reported alcohol intake measured by TLFB and analyzed by:
  - Mean grams alcohol per day
  - Number of drinking days
  - Number of drinks per drinking days
  - Number of abstinent days
- Total score of Alcohol Use Identification test AUDIT
- Alcohol Craving measured by Visual analogue scale (VAS)
- Nicotine use measured by nicotine saliva marker cotinine
- Temporal experience of pleasure scale (TEPS)
- The continuous performance test + Activity test (CPTA)
- To assess the relationships between the above described outcome measures and plasma drug concentrations of BUP and VAR measured at visit 4 and 6

8 STATISTICAL AND ANALYTICAL PLANS

8.1 General principles

The SAS® software, version 9.2 or higher, will be used to produce all of the statistical outputs. All efficacy and safety variables will be presented using descriptive statistics and graphs as appropriate. Safety measures will be summarized descriptively using the safety analysis set. Continuous variables will be presented with descriptive statistics (n, mean, standard deviation [SD], median, min, max), within treatment group. Categorical variables will be summarized by treatment group in frequency tables (number of patients and percentage). The denominators for all percentages will be

Transaktion 09222115557481461024 Signerat MP

defined as the number of non-missing values for each variable within the analysis set. Percentages will be presented with 1 decimal place.

8.1.1 Visit windows

Timing of visit window are described in the CSP (Version 1, 2018-02-09).

8.1.2 Baseline

Baseline values will be collected at the screening visit (Visit 1).

8.1.3 Treatment period

The steady-state active treatment period is defined as visit 4 until visit 8 and efficacy analysis will be evaluated during this period.
For secondary outcome measures, the treatment period is also defined as visit 4 until visit 8.

Data from visit 2 and visit 4 is excluded because of dose titration and time to steady state. Data from visit 9 is excluded due to a higher expected drop-out rate.

8.1.4 Follow-up Period

The follow-up period begins the day after the treatment period ends and ends on the date of the last planned telephone follow-up contact (visit 9 (EoT)+30days). The follow-up period is targeted to last for 30 days.

8.1.5 Fel! Ogiltig självreferens i bokmärke.**Multiplicity**

Regulatory agency will consider only one specific primary objective and since the two primary endpoints fulfill regulatory requests independently and separately, hence no adjustment for multiplicity has been done.

As testing between treatments are performed in a hierarchical fashion, no adjustment for multiple testing has been done.

8.1.6 Handling of missing data

In a blind data review, missing data will be assessed, see section 8.2.
Since PEth is expected to decline over time for patients on combination dose,
we believe that the most important measurement for effect is visit 4. In order to get a proper evaluation of the active treatment period a suitable approach for evaluation of PEth, would be to exclude patients from analysis if either of the following criteria are met:

- Patient is absent from scheduled visit at visit 4
- Patient is absent from more than two scheduled visits during the steady-state active treatment period (visit 4 visit 8)

This missing data policy is considering that earlier data will be expected to have higher values, and hence, they being omitted will increase power.

Considering HDD as primary objective, previously found, in the varenicline study (deBejczy et al 2015) a drinking pattern could be detected, and it was found that the last

Transaktion 09222115557481461024 Signerat MP

observation carried forward imputation method for missing data was not a suitable imputation method.
We believe that Missing Not At Random MNAR can be assumed.

Missing data for evaluation of HDD will be handled in the same way as in the earlier cited varenicline study. The technique for imputing data is as follows: If a subject is missing data from one day then a weighted average of that subject's values from the two corresponding weekdays before and after this, if available, will be imputed for that subject, with weights 2 assigned for the closest corresponding days and weights 1 assigned for the second closest corresponding days. *Example: Subject A is missing data from Wednesday of week 5 of the active treatment period. The subject has the following available data on the two Wednesdays before this, i.e. weeks 3 and 4, as well as the two Wednesdays after this, i.e. weeks 6 and 7: The Wednesday of week 3 was a heavy drinking day (HDD), the Wednesday of week 4 was not a HDD, the Wednesdays of weeks 6 and 7 were not HDD's. Then the value to impute for the missing Wednesday will be (1*1 + 2*0 + 2*0 + 1*0)/6 = 1/6 = 0,167. Subject B is missing the Thursday of week 3 of the active treatment period. This is the first week with post baseline measurements, so there will be no values to impute from weeks before. But this subject has values from the two following Thursdays: Thursday of week 4 is a heavy drinking day and the Thursday of week 5 is not. Thus the value imputed for subject B will be (2*1 + 1*0)/3 = 0,667.*
If a subject drops out of the study then the remaining days for that subject will be imputed in one of the following two ways, depending on how long the subject has participated in the study before the drop out:

1) If a subject has attended 4 visits or fewer and then drops out of the study, then the subject's baseline proportion of HDD's will be carried forward. In other words the baseline observation carried forward (BOCF) method will be used. The rationale for this is that the subject has received at the most two weeks of active treatment: week 1 which is the dose escalation week and week 2, during which steady state is expected to be reached. Thus it is not reasonable to expect that the subject will have received a sustainable benefit from the treatment. It is more likely that the subject's alcohol consumption will return to the baseline level.
2) If a subject has attended at least 5 visits and drops out of the study, then the last observed seven days for that subject will be carried forward, i.e. the last observation carried forward (LOCF) method will be used. If the last observed seven days are not complete, they will first be completed according to Table of imputation method in the Appendix. The rationale for using LOCF for these subjects is that it may be reasonable to assume that they have derived a sustainable benefit from the treatment.

For other variables missing data is considered as missing and no imputation will be done.

8.1.7 Handling of outliers

If outliers are identified during the evaluation or in the descriptive analysis, they will be listed and evaluated.

Transaktion 09222115557481461024 Signerat MP

8.1.8 Patient disposition

A summary table, by treatment arm and overall, will be presented for the number of patients who have been:

- Enrolled
- Randomized
- Received at least one dose of randomized IP
- Did not receive treatment
- Completed the study
- Prematurely discontinued the study (along with the categorized reason for discontinuation)

The number of patients included in each of the analysis sets will also be tabulated, as well as reasons for exclusion from each analysis set.

8.1.9 Protocol Deviations

A list of important protocol deviations will be finalized prior to study un-blinding. The number and percentages of patients with at least one important protocol deviation will be summarized by treatment arm and overall. The number and percentages of patients within each type of protocol deviation will be provided as well.

A listing of all patients with any important protocol violations/deviations will be provided for them mITT population.

8.2 Blind data review

A blind data review will be performed while data is still double blind.

The number of missing data and data quality will be assessed.

Number of valid data for HDD during follow up period is screened. Number of patients excluded (see Section 8.1.6) is reported. If the drop-out rate is considered high, analyses criteria will be reviewed.

8.3 Analysis of primary efficacy variable

Primary efficacy variables will be analyzed according to mITT and will be summarized descriptively. Analysis will be presented in tables presenting estimates of the coefficients, their 95 % confidence intervals and p-values.
Kaplan-Meier figures will be provided as appropriately.

Primary efficacy variables will also be analyzed with the same approach described above using the PP analysis set.

Sensitivity analyses will be done on the mITT population in order to study the robustness of the analysis with respect to handling of missing data.

Other post-hoc tests will be used if needed and as appropriate.

Transaktion 09222115557481461024 Signerat MP

8.3.1 PEth

An analysis with regards to PEth, will be performed using an analysis of covariance (ANCOVA) using the mean value of PEth (visit 4 -visit 8) within patient, as the dependent variable.

Adjustments for following covariates will be performed: treatment group, center, nicotine use (Yes/No) and family history positive, FHP (Yes/No).
The ANCOVA model will have fixed effects for treatment and center and subject, nicotine use and FHP will be entered as random effect.
Baseline PEth will be used as a covariate in the analysis.

$$mean\ of\ PEth(visit4 - visit8) = \beta_0 + \beta_1 \cdot PEth(baseline) + \beta_2 \cdot Treatment + \beta_3 \cdot Smoking + \beta_4 \cdot FHP + \beta_5 \cdot dummy_1 + \beta_6 \cdot dummy_2 + \beta_7 \cdot dummy_3$$

β_0 is a constant

β_1 is the coefficient for the baseline Peth value (screening visit)

β_2 is the difference between treatment groups controlled for the other factors in the model

β_3 is the coefficient for smoking (Yes/No)

β_4 is the coefficient for FHP (Yes/No)

β_5, β_6 and β_7 are dummy variables for center (see coding in the table in the next Section)

The ANCOVA analysis compares the mean values of the variable in question for the different treatment groups hierarchically:

Step1: H_{01}: A+B = $placebo_A$+$placebo_B$

H_{11}: A+B ≠ $placebo_A$+$placebo_B$

If significant difference proceed to step2.

Step2: H_{02}: A+ $placebo_B$ = $placebo_A$+$placebo_B$

H_{12}: A+ $placebo_B$ ≠ $placebo_A$+$placebo_B$

If significant difference proceed to step3.

Step3: H_{03}: A+B = A+$placebo_B$

H_{13}: A+B ≠ A+$placebo_B$

If significant difference proceed to step4.

Step4: H_{03}: A+B = B+$placebo_A$

H_{13}: A+B ≠ B+$placebo_A$

Standard analyses of the residuals will be performed to evaluate that the assumptions for ANCOVA models are fulfilled. If assumptions of normality are not met, a non-parametric analysis will be made to study the robustness of the ANCOVA analysis.

Transaktion 09222115557481461024 Signerat MP

8.3.2 Heavy Drinking Days (HDD)

An analysis of covariance (ANCOVA) will be performed for the primary variable where the number of of HDD's as a total over visit 4 - visit 8 by 14 days, will be used as dependent variable. Adjustment for the covariates treatment group, smoking (Yes/No), family history positive, FHP, (Yes/No) and center will be performed.

Baseline alcohol consumption will be used as a covariate in the analysis.

HDD baseline for efficacy= definition TLFB30 by 14 days

The ANCOVA model will have fixed effects for treatment and center and subject, nicotine use and FHP will be entered as random effect. The structure of the model for the primary variable will thus be as follows:

$$HDD(visit\ 4 - visit\ 8) = \beta_0 + \beta_1 \cdot HDD(baseline) + \beta_2 \cdot Treatment + \beta_3 \cdot Smoking + \beta_4 \cdot FHP + \beta_5 \cdot dummy_1 + \beta_6 \cdot dummy_2 + \beta_7 \cdot dummy_3$$

β_0 is a constant

β_1 is the coefficient for the HDD baseline (TLFB30) by 14days

β_2 is the difference between treatment groups controlled for the other factors in the model

β_3 is the coefficient for smoking (Yes/No)

β_4 is the coefficient for FHP (Yes/No)

β_5, β_6 and β_7 are dummy variables for center (see coding in table below)

Tabell 1. Dummy variables for center

**Center/Dummy**	**D1**	**D2**	**D3**
Site1	0	0	0
Site2	1	0	0
Site3	0	1	0
Site4	0	0	1
Site5	1	1	0
Site6	0	1	1

The ANCOVA analysis compares the mean values of proportions of the variable in question for the different treatment groups in a hierarchical fashion as described in section 8.2.1.

Transaktion 09222115557481461024 Signerat MP

Standard analyses of the residuals will be performed to evaluate that the assumptions for ANCOVA models are fulfilled. If assumptions of normality are not met, a non-parametric analysis will be made to study the robustness of the ANCOVA analysis.

8.4 Analysis of secondary efficacy variable

Secondary outcomes will be analyzed according to mITT and PP.

8.4.1 Additional alcohol markers

Additional alcohol markers CDT and GGT will be collected at every visit and will be analyzed as a mean over the steady state treatment period (visit 4 – visit 8) in a similar manner as B-PEth, and compared to baseline. Additional analyses of specific time-points will be carried out. Data will be analyzed descriptively and statistical comparisons between treatments will be performed by a t-test or Wilcoxon test, depending of distribution and sample size.

8.4.2 Subjective alcohol consumption

For secondary objectives, Time Line Follow Back (TLFB) is the measurement of subjective alcohol consumption. Mean grams alcohol per day, number of drinking days, number of abstinent days, number of drinks per drinking day, will be evaluated over the active treatment period (visit 4 - visit 8).

Baseline of TLFB will be derived using TLFB30/TLFB14 at screening.

Data will be analyzed descriptively and statistical comparisons between treatments will be performed by a t-test or Wilcoxon test, depending of distribution and sample size.

8.4.3 AUDIT

AUDIT will be measured as a total score at visit 1 and at visit 8. Change from baseline will be calculated. Data will be analyzed descriptively and statistical comparisons between treatments will be performed by a t-test or Wilcoxon test, depending of distribution and sample size.

8.4.4 Alcohol craving (VAS)

Alcohol craving will be measured by Visual analogue scale (VAS) using the measures craving, motivation, temper. Data will be analyzed as a mean over active treatment period (visit 4 - visit 8) and for specific time-points. Change from baseline will be calculated. Data will be analyzed descriptively and statistical comparisons between treatments will be performed by a t-test or Wilcoxon test, depending of distribution and sample size.

8.4.5 Nicotine use

Nicotine use measured by nicotine saliva marker cotinine and will be measured at visit 2 and visit 8, change from baseline will be calculated. Data will be analyzed descriptively and statistical comparisons between treatments will be performed by a t-test or Wilcoxon test, depending of distribution and sample size.

© Statistikkonsulterna

Transaktion 09222115557481461024 Signerat MP

8.4.6 Temporal experience of pleasure scale (TEPS)

Temporal experience of pleasure scale (TEPS) will be measured at visit 1 and visit 8, change from baseline will be calculated. Data will be analyzed descriptively and statistical comparisons between treatments will be performed by a t-test or Wilcoxon test, depending of distribution and sample size.

8.4.7 Opatus®CPTA

Opatus®CPTA consists of continuous measurements that are weighted into three composite variables corresponding to activity, attention and impulsivity.

Opatus®CPTA will be measured at visit 1 and visit 8 and change from baseline will be calculated. Appropriate statistical testing will be performed.

8.4.8 Interaction of alcohol and nicotine consumption

Interaction between alcohol and nicotine consumption will be analyzed.

8.5 Subgroup for exploratory analysis

1) Exploratory analyses will be performed on the impact of demographic data (e.g. gender) on the above described endpoints
2) Exploratory analyses will be performed to assess the relationship between the above primary and secondary outcome measures
3) Exploratory analyses will be performed to assess the effects of the above primary and secondary outcome measures in relation to blood drug concentrations of BUP and VAR
4) Subgroup analysis will be performed on gender
5) Subgroup analysis will be performed on heredity, as defined by family history positive (FHP)
6) Subgroup analysis will be performed on consumption levels at screening, as defined by WHO consumption levels categories; 0-40 g/day, 40-60 g/day, 60-100 g/day and over 100 g/day.
7) Subgroup analysis will be performed on DSM diagnostic criteria severe AUD at screening, as defined by 6 or more criteria
8) Subgroup analysis will be performed on early debute of alcohol consumption (age 12 and below) as a proxy for early onset AUD
9) Subgroup analysis will be performed on responders/non-responders as defined by 1) a downward shift of two WHO consumption level categories as measured by TAC, compared to baseline 2) a 50% reduction in PEth levels compared to baseline
10) Subgroup analyses will be performed on Early Responders, as defined by 1) a 50% or more reduction in PEth levels alcohol 2) a 50% more reduction in HDD 3) a 50% reduction in TAC between visit 1 (screening) and visit 2 (randomization/start IMP).

Transaktion 09222115557481461024 Signerat MP

11) Subgroup analysis will be performed on level of ADHD symptoms, as defined by ASRS scores 17 or more at screening.

8.6 Sensitivity analysis

Sensitivity analyses of primary outcome will be performed with regards to both PEth and HDD. A complete case analysis will be performed using all patient with a full set of study data with regards to PEth and HDD.

Will be performed regarding site effects and regarding Last Observation Carried Forward (LOCF) for the variable TLFB to determine possible influence on results of imputation vs non-imputed data.

Depending on results from the blind data review, additional sensitivity analysis of primary outcome may be identified.

8.7 Summary of efficacy analysis

Primary/Secondary (Analysis set)	Variable	Unit	Statistical methods
Primary (mITT/PP)	PEth (blood levels)	μmol/l	Descriptive statistics, ANCOVA
Primary (mITT/PP)	Number of heavy drinking days (HDD)by 14 days	#/14days	Descriptive statistics, ANCOVA
Secondary (mITT/PP)	Additional blood alcohol markers measured in blood (CDT, GT)	%, μkat/l	Descriptive statistics, Statistical testing
Secondary (mITT/PP)	TLFB<br>Mean grams alcohol per day<br>Number of drinking days<br>Number of abstinent days<br>Number of drinks per drinking days	 gram<br>days<br>days<br>days	Descriptive statistics, Statistical testing
Secondary (mITT/PP)	Concentration of study medication in plasma blood<br>Bupropion,<br>Varenicline	 ng/ml<br>ng/ml	Descriptive statistics, Statistical testing
Secondary (mITT/PP)	AUDIT	total score	Descriptive statistics, Statistical testing

Transaktion 09222115557481461024 Signerat MP

Secondary (mITT/PP)	Alcohol craving (VAS)	score	Descriptive statistics, Statistical testing
Secondary (mITT/PP)	Cotinine in blood	ng/ml	Descriptive statistics, Statistical testing
Secondary (mITT/PP)	Temporal experience of pleasure scale (TEPS)	Total score	Descriptive statistics, Statistical testing
Secondary (mITT/PP)	CPTA® (Opatus)		Descriptive statistics, Statistical testing

9 INTERIM ANALYSIS

NA

10 CHANGES OF ANALYSIS FROM PROTOCOL

NA

11 REPORTING AND PUBLICATION OF RESULTS

According agreement with Addiction Biology Unit

© Statistikkonsulterna

Transaktion 09222115557481461024 Signerat MP

12 APPENDIX

The table of imputation uses two example subjects to illustrate how the last observed seven day will be carried forward for the subjects which have attended at least 5 visits before dropping out of the study. Example subject two has two missing days during the last observed week: Friday and Tuesday. The imputed value for Friday will be the weighted average of the two preceding Fridays: (1*1+2*1)/3=3/3=1. The imputed value for Tuesday will be (2*1+1*0)/3=0,667. When these two gaps have been filled out, the last seven days will be carried forward.

Table 3 Table of imputation

Subject	Week	Weekday	HDD	Imp HDD
...	...	...	...	...
1	5	Mon	0	
1	5	Tue	0	
1	5	Wed	0	
1	5	Thu	0	
1	5	Fri	1	
1	5	Sat	1	
1	5	Sun	0	
1	6	Mon	0	
1	6	Tue	1	
1	6	Wed	0	
1	6	Thu	1	
1	6	Fri	1	
1	6	Sat	1	
1	6	Sun	1	
1	7	Mon	0	
1	7	Tue	0	
1	7	Wed	0	
1	7	Thu	Drop out	1
1	7	Fri	Drop out	1
1	7	Sat	Drop out	1
1	7	Sun	Drop out	1
1	8	Mon	Drop out	0
1	8	Tue	Drop out	0
1	8	Wed	Drop out	0
1	8	Thu	Drop out	1
1	8	Fri	Drop out	1
1	8	Sat	Drop out	1
1	8	Sun	Drop out	1
...	...	...	...	...
2	5	Mon	0	
2	5	Tue	1	
2	5	Wed	1	

Transaktion 09222115557481461024 Signerat MP

2	5	Thu	0	
2	5	Fri	1	
2	5	Sat	1	
2	5	Sun	0	
2	6	Mon	0	
2	6	Tue	0	
2	6	Wed	0	
2	6	Thu	0	
2	6	Fri	1	
2	6	Sat	1	
2	6	Sun	0	
2	7	Mon	0	
2	7	Tue	1	
2	7	Wed	0	
2	7	Thu	1	
2	7	Fri		1
2	7	Sat	1	
2	7	Sun	1	
2	8	Mon	0	
2	8	Tue		0,666667
2	8	Wed	1	
2	8	Thu	Drop out	1
2	8	Fri	Drop out	1
2	8	Sat	Drop out	1
2	8	Sun	Drop out	1
2	9	Mon	Drop out	0
2	9	Tue	Drop out	0,666667
2	9	Wed	Drop out	1
2	9	Thu	Drop out	1
2	9	Fri	Drop out	1
2	9	Sat	Drop out	1
2	9	Sun	Drop out	1
...	...	...	...	...

Transaktion 09222115557481461024 Signerat MP

Verifikat

Transaktion 09222115557481461024

Dokument

COMB SAP final 15nov2022
Huvuddokument
19 sidor
Startades 2022-11-15 08:22:55 CET (+0100) av Magnus Pettersson (MP)
Färdigställt 2022-11-15 08:23:57 CET (+0100)

Signerande parter

Magnus Pettersson (MP)
Statistikkonsulterna
magnus.pettersson@statistikkonsulterna.se
+460703731297

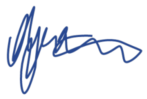

Signerade 2022-11-15 08:23:57 CET (+0100)

Detta verifikat är utfärdat av Scrive. Information i kursiv stil är säkert verifierad av Scrive. Se de dolda bilagorna för mer information/bevis om detta dokument. Använd en PDF-läsare som t ex Adobe Reader som kan visa dolda bilagor för att se bilagorna. Observera att om dokumentet skrivs ut kan inte integriteten i papperskopian bevisas enligt nedan och att en vanlig papperutskrift saknar innehållet i de dolda bilagorna. Den digitala signaturen (elektroniska förseglingen) säkerställer att integriteten av detta dokument, inklusive de dolda bilagorna, kan bevisas matematiskt och oberoende av Scrive. För er bekvämlighet tillhandahåller Scrive även en tjänst för att kontrollera dokumentets integritet automatiskt på: https://scrive.com/verify
