# Supplementary material for: A randomized, double-blind, placebo-controlled, multicentre trial on the efficacy of varenicline and bupropion in combination and alone for treatment of alcohol use disorder: Protocol for the COMB study
Source: PLoS One. 2024 Jan 11;19(1):e0296118. doi: 10.1371/journal.pone.0296118 (PMC10783749; doi:10.1371/journal.pone.0296118)
Supplement: S4 File — (PDF) [file pone.0296118.s004.pdf]

Machine Translated by Google

MONITORING PLAN FOR CLINICAL DRUG TRIAL COMB-BO8

Study protocol The
monitoring plan refers to Protocol COMB-BO8, Version Number: 2, Lidö & deBejczy, 2018-06-14.
EudraCT Number: 2018–000048-24 and from August 2019 Protocol Version 3, Lidö & deBejczy, 2019-05-20

Sponsor
The study is an academic investigator-initiated drug study where the sponsor and principal investigator is Bo Söderpalm, Addiction Clinic, Sahlgrenska University Hospital, Västra Götaland Region. The study is mainly financed by the Swedish Research Council.

Study title
A randomized, double-blind, placebo-controlled multicenter trial on the efficacy of varenicline and bupropion in combination and alone, for treatment of alcohol use disorder. Abbreviation ´COMB study'

Purpose The COMB study is a Phase II study, which tests the effects of varenicline and bupropion, in combination and separately, in the treatment of alcohol dependence. The study has two primary outcome measures: 1) the biological marker for alcohol consumption phosphatidylethanol measured in blood and 2) heavy drinking days (HDD), measured via the Time Line Follow Back (TLFB) procedure

Volume
A total of 380 research subjects are to be included in the study. These are randomized equally to 4 treatment arms (95 in each arm). The active phase is 13 weeks (91 days) and consists of 9 study visits. The first 3 study visits take place once a week (including the screening visit).
The remaining 6 visits take place every 14 days.

Study center
Around 6 centers will participate in the study, Västra Götaland region (Addiction clinic, Sahlgrenska inversitetssjukhuset and Borås), Halland region, Linköping region, Skåne region and Stockholm County Hospital Area. This monitoring plan will be followed at all centers.

Timetable
Active phase is estimated at 3-4 years. The study starts in March 2019 and is expected to end in December 2023.
Follow-up work until June 2024.

Monitoring plan
The monitoring plan follows the principles of ICH-GCP for monitoring drug trials

The study will monitor the before, during and after the clinical phase.
-Pre-study visit before the start of studies. On-site and remote monitoring -Monitoring visits during the course of the study and remote monitoring -Closing visits

Machine Translated by Google

Before the monitoring visit,

Monitor contacts the responsible examiner well in advance of the visit to ensure that CRFs are filled in, that the examiner's folder and journal entries are available and that time is set aside for the visit. A confirmation letter will be sent to the examiner before the visit.

Monitoring visit Before

the study. Monitor will, depending on the time, attend the initiation visit together with the Sponsor's study team

The first monitoring visit is booked as soon as possible at each site after the first patient has been screened.
The closing visit will take place as soon as possible after Data Base Lock.
The frequency of monitoring visits between the initiation meeting and the closing visit may vary between sites depending on need. Each visit is estimated to be 3-6 hours on site over 1-2 days. Between monitoring visits, the eCRF is checked continuously and questions are asked if necessary.

Risk-based source data verification and monitoring of essential documents

100% monitoring of critical variables.

ÿ 100% monitoring occurs for the first 2 subjects at each study center. The monitoring includes all data in the eCRF, i.e. all visits/forms/variables, incl/excl criteria, informed consent and social security number, study-specific samples, screening and randomisation numbers, adverse events, visit dates and journal entries.

ÿ 100% monitoring occurs for 20% of subjects. The volume is estimated at approximately 76 pcs including the first 2 subjects. The randomization numbers to be fully monitored are randomly selected by digital system in electronic CRF.

ÿ For the remaining subjects, monitoring of: • Informed consent •
Inclusion and exclusion criteria • Primary outcome
variables TLFB and B-PEth at all visits • Adverse events
• Pharmacokinetic sampling at study visits 4 and 6

ÿ 100% drug accountability (pill count) i.e. delivery and submission of study drugs, for all study participants at all visits.

ÿ Additional monitoring in addition to the above plan if deficiencies exist.

ÿ Monitoring of examiner files, all study logs and essential documents in addition to CRF, including delegation list, CV and GCP authorization.

ÿ All Serious Adverse Events (SAE) must be monitored, including the reporting from the Sponsor to The Swedish Medicines Agency within the prescribed time frame.

The study uses electronic CRF. The system makes the random selections and flags for the monitor which subjects are to be fully monitored according to the plan and which forms/variables are to be monitored for the other subjects.

Machine Translated by Google

Monitoring report After each monitoring visit, a report is written. The report is written in accordance with ICH/GCP guidelines and must, among other things, contain a summary of deviations, changes, improvements or remarks made during the visit. Monitor then writes a follow-up letter which is sent to the study centre.

Confidentiality bond

The monitor is responsible for ensuring that a confidentiality agreement is drawn up and signed by the records manager at each site before the first monitoring visit

Archiving

Archiving will take place at the trial site for 10 years.
