# Supplementary material for: A randomized, double-blind, placebo-controlled, multicentre trial on the efficacy of varenicline and bupropion in combination and alone for treatment of alcohol use disorder: Protocol for the COMB study
Source: PLoS One. 2024 Jan 11;19(1):e0296118. doi: 10.1371/journal.pone.0296118 (PMC10783749; doi:10.1371/journal.pone.0296118)
Supplement: S1 Table — (PDF) [file pone.0296118.s006.pdf]

S1 Table. Study Sites participating in the COMB Study.

**Principal Investigator, Site 1**<br>**Sponsor**	Bo Söderpalm, MD,PhD<br>Professor of Psychiatry	Addiction Biology Unit (ABU), Sahlgrenska University Hospital, Gothenburg, Sweden
**Investigator, Site 2**	Markus Heilig, MD, PhD<br>Professor of Psychiatry	Center for Social and Affective Neuroscience (CSAN), Linköping University Hospital, Linköping, Sweden
**Investigator, Site 3**	Johan Franck, MD, PhD<br>Professor of Psychiatry	Stockholm Centre for Dependency Disorders (SLSO), Stockholm, Sweden
**Investigator, Site 4**	Anders Håkansson, MD, PhD<br>Professor of Psychiatry	Malmö Addiction Center, Malmö, Sweden

The study centres are addiction and dependency research units. The investigators are all professors in psychiatry and experienced clinicians in the field. All study personnel are proficient in Good Clinical Practice (GCP) and study procedures.
